# A Perspective into *Operando* Methods for Probing Catalytic Interfaces

**DOI:** 10.1021/acs.jpclett.5c02150

**Published:** 2025-12-30

**Authors:** Olivia J. Alley, Yue Liu, Francesca M. Toma

**Affiliations:** † Chemical Sciences Division, 1666 Lawrence Berkeley National Laboratory, Berkeley, California 94720-8099, United States; ‡ Institute of Functional Materials for Sustainability, Helmholtz-Zentrum Hereon, Kantstrasse 55, 14153, Teltow, Brandenburg, Germany; § Faculty of Mechanical and Civil Engineering, Helmut Schmidt University, Holstenhofweg 85, 22043, Hamburg, Germany

## Abstract

Rational design of electrocatalysts for (photo)­electrochemical (PEC) processes like hydrogen and oxygen evolution and CO_2_ reduction reactions is aided by the recent improvements in capabilities of *operando* measurements, where morphology, composition, and/or function are probed during active catalysis. Through *operando* microscopy and spectroscopy, structure, catalytic microenvironment, oxidation state, adsorbates, and products can be measured to gain a better understanding of catalyst behavior and suggest possible improvements. Visualizing evolving catalyst morphologies, surface compositions, and electrochemical behavior also helps address many fundamental research questions for a better understanding of catalytic mechanisms. Correlating morphology with chemical identity or functional behavior using a variety of innovative microscopy methods is particularly promising for guiding development of next generation catalysts, and there are also many recent examples of using AI and robotics tools to innovate and speed development. In this Perspective, advances made over the past few years in *operando* imaging of catalysts relevant to solar fuels will be explored, followed by an outlook on technological developments in instrumentation, sample design, and computational power that may be applied to this field.

## Graphical abstract

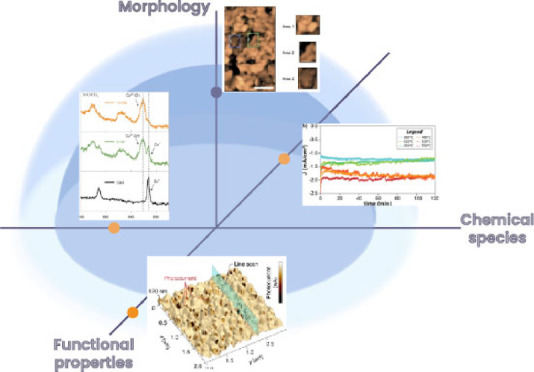

More than a century passed between Giacomo Ciamician’s 1912 article in *Science* on the potential of solar energy for creating a more prosperous and equitable world, and Barack Obama’s 2017 policy article in *Science* on the urgent need for replacing fossil fuel energy with carbon-free sources.^1,2^ It has become clear that there is no crisis of supply of fossil fuels, as Ciamician feared, but rather a crisis of global climate change brought about by their consumption. Energy storage is a major topic when thinking about moving to clean energy because of the intermittent nature of solar and wind. While batteries can be used for storage in many applications, they lack scalability and are difficult to recycle. Hydrogen and liquid fuels like methanol, ethanol, or ammonia, synthesized electrochemically using clean energy and renewable reagents are a scalable and flexible energy storage solution.

There are three common routes to make solar fuels. All of them rely on a light-absorbing semiconductor that converts solar energy into charge carriers driving chemical reactions. In PhotoVoltaic–ElectroChemical (PV-EC) devices, the semiconductor is part of the photovoltaic (PV) module, which is externally coupled (wired) to an electrochemical cell. The PV-generated electricity powers the fuel-forming reactions. In PhotoElectroChemical (PEC) solar fuel generation, the semiconductor is integrated into a photoelectrode that is in direct contact with the electrolyte. Upon illumination, photogenerated charge carriers in the semiconductor directly reach the electrolyte and drive oxidation and reduction reactions. In this architecture, catalysts and protective layers are often incorporated into the photoelectrode and interfaced with the semiconductor to improve reaction selectivity and mitigate challenges related to stability and losses. Lastly, in PhotoCatalysis (PC), the semiconductor is in particulate form or deposited on a substrate, and both oxidation and reduction reactions occur directly at its surface upon illumination. PV-EC has the benefit of reduced corrosion challenges and significantly higher current densities, while PEC is being studied for the kinetic boost the electrochemical reactions have from their proximity to the heat generated at the photoabsorber. PC shows promise as well for its theoretical scalability, yet the efficiency is still low. For each method, developing high quality electrocatalysts is central to moving solar fuels from the lab to the market.

Across these approaches, solar-to-fuel conversion relies on key interfaces that couple light absorption, charge separation, and chemical reactivity. In this context, photoelectrodes are semiconductors that harvest light to generate charge carriers driving these reactions, and that may also exhibit catalytic activity. More commonly, they are coupled with metallic or oxide electrocatalysts that facilitate charge transfer at the semiconductor–electrolyte interface and enhance activity and stability. Considering this full spectrum of interfaces provides a unifying framework to understand how light-driven and electrochemical processes interconnect and emphasizes the importance of characterizing interfacial phenomena under realistic operating conditions.

For scaling up solar fuel generation via PEC systems, the photoelectrode architecture must (i) effectively decrease reaction overpotentials, (ii) maintain high solar-to-fuel conversion stability and faradaic efficiency, and (iii) be made from inexpensive, earth-abundant materials. To date, it has not been possible to fulfill all three criteria simultaneously. One of the main challenges, shared with the field of electrocatalysis, is that many of the highest-performing PEC systems rely on photoelectrodes that incorporate rare platinum group metals (PGMs) as metallic catalysts. When interfaced with the semiconductor, the catalyst has the important function to help drive charge carriers at the photoelectrode/electrolyte interface and improve reaction kinetics. For solar fuels to scale, PGMs should either be replaced, necessitating an understanding of what makes them so effective, or made more durable and used more efficiently through atomic dispersion or alloying strategies. The increasing complexity of these semiconductor–catalyst interfaces underscores the need to understand surface evolution during catalysis. Beyond scalability, many complex solar fuel reactions still suffer from low catalytic efficiency or selectivity, even with the best available materials, and research continues to focus on identifying pathways for improvement. Finally, improving the economic and environmental feasibility of solar fuels requires highly durable photoelectrodes. However, many current technologies still operate for only a few days or weeks before corrosion leads to performance degradation. Aligned with these goals, a fundamental understanding of the catalytic interface under realistic operating conditions is crucial.

A distinction is often drawn in the literature between *operando* and *in-situ* measurements, depending on how closely the measurement conditions approach true operating conditions. It is commonly accepted in the community that the term *operando* should more specifically refer to measurements that probe morphological, chemical speciation, or other physicochemical properties under (or close to) simulated reaction conditions while simultaneously measuring (photo)­electrocatalytic performance. In many, if not all, of the cases presented here, significant challenges remain in probing (photo)­electrocatalysts under truly representative *operando* conditions. This ambiguity between *in-situ* and *operando* definitions can directly affect how structure–function relationships are interpreted, as data collected under partially relevant conditions may not capture transient states or realistic surface compositions present during operation. Therefore, careful reporting and discussion of the experimental parameters become essential for assessing the true mechanistic implications. We acknowledge that both *in-situ* and *operando* techniques, despite their limitations, currently represent the most effective tools available to the community for gaining insights into these complex catalytic systems. As a result, the distinction between *in-situ* and *operando* becomes increasingly blurred, especially for (photo)­electrochemical systems. Beyond terminological precision, best practice requires authors to explicitly report all relevant measurement conditions, including temperature, potential, electrolyte composition, and illumination intensity, and to indicate whether functional properties were simultaneously monitored. Here, to maintain clarity in the discussion, we only use the word *operando* while explicitly stating the operating conditions for each measurement.

In Figure 1, we provide a visualization of the *operando* methods discussed in this paper, with each axis corresponding to morphological, chemical, or functional data, and the space divided into three regions according to measurement conditions, with conditions closest to true *operando* conditions closer to the graph’s origin. The innermost dark blue hemisphere represents measurements done in a liquid or electrochemical cell, with the sample immersed in solution. The larger light blue hemisphere represents measurements that were done at atmospheric pressure, but not in solution, and finally the area outside the light blue hemisphere has methods which have pressure less than 1 atmospheric pressure, but still above the standard operating conditions of the analytical technique. This is important because many analytical techniques rely on a controlled atmosphere or high vacuum, and for these techniques careful process development is required to do measurements under even moderate pressure. This type of measurement is also useful to study the interaction of a catalyst surface with a gas.

**Figure 1:**
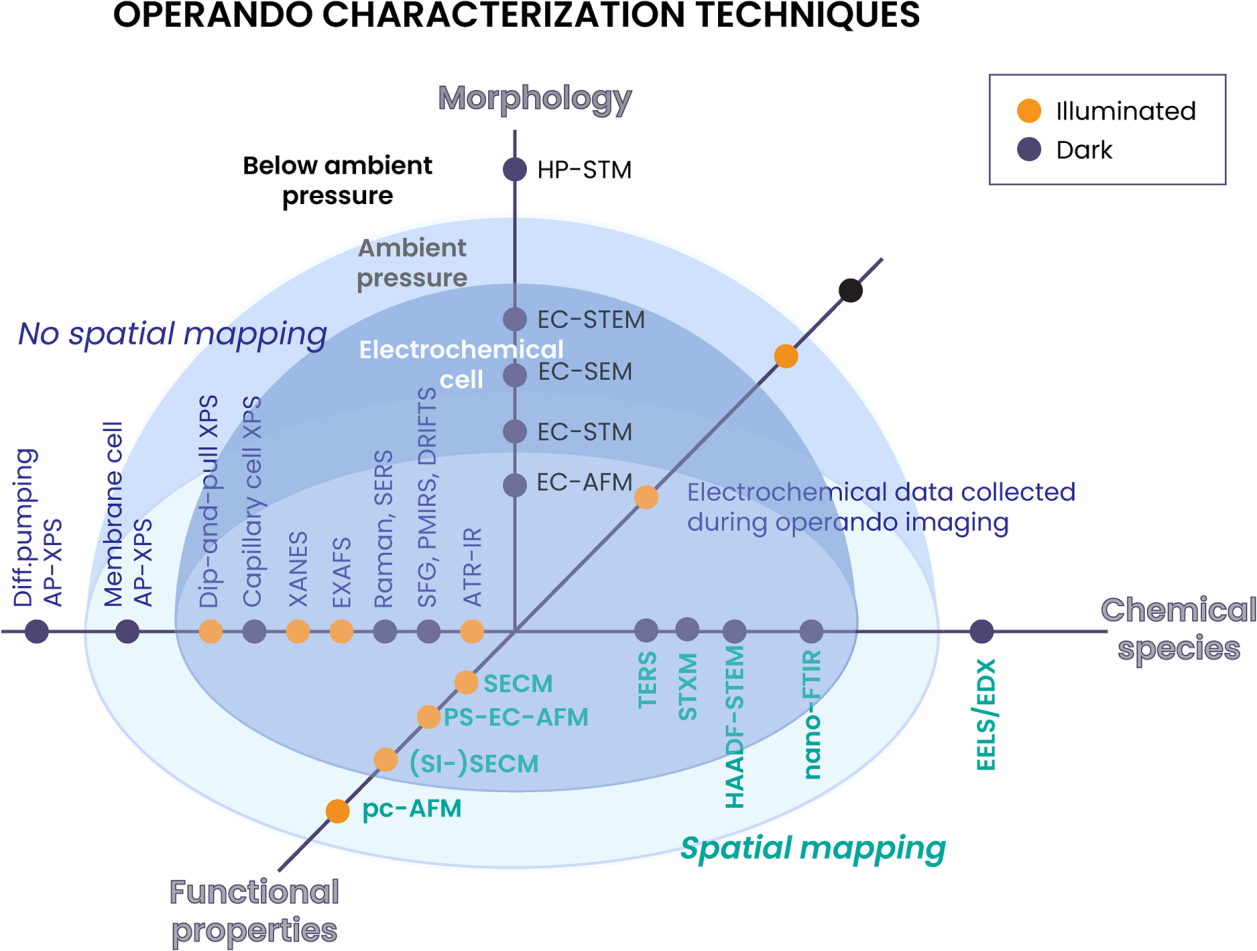
Visualization of the methods described in the perspective, organized along the axes by their probing significance (i.e., morphological, chemical, and functional information) and spatial resolution capability. The grouping in the hemispheres indicates measurements done in a liquid cell (dark blue), or at atmospheric pressure but not in an electrochemical cell or in solution (light blue), or below 1 atmospheric pressure (outside the light blue hemisphere).

The graph’s origin also separates the methods into those that do or do not produce a spatially resolved map, with the former corresponding to the front right quadrant methods (‘spatial mapping’) and the latter to the back left quadrant (‘no spatial mapping’). In Figure 1, the axis labeled ‘chemical species’ shows methods which identify evolving species present during catalysis, while the axis labeled ‘functional properties’ shows methods that provide information on the catalyst’s functional properties, including conductivity, kinetics, or potentials. Finally, the axis labeled ‘morphology’ has methods that show different aspects of morphology. Photoelectrochemical/photocatalytic measurements done in the presented examples under illumination are distinguished by yellow points, while electrochemical measurements done without illumination are black points.

Recent advances in methods and instrumentation facilitate *operando* imaging to visualize reaction intermediates, catalytic center bonding, surface adsorbates, transient oxide species, and morphological changes of electrocatalysts for a better understanding of activation and catalytic processes. While *ex-situ* characterization can track stable corrosion products, oxidation state changes, and permanent morphological evolution, and these can be correlated with functionality changes,^3,4^ illumination and electrochemical conditions can both create transient shifts in surface conditions. Time resolved imaging is particularly relevant for observing corrosion and particle growth. Therefore, *ex-situ* and *operando* techniques are complementary in an effort to understand and optimize solar fuel catalysts, and often multiple methods are used together to develop a comprehensive understanding of a catalyst’s corrosion processes, chemical and morphological transformations, kinetics, or reaction intermediates. For example, in our group, we used ambient-pressure X-ray photoelectron spectroscopy (AP-XPS) to determine the corrosion mechanisms present in the photoelectrochemical carbon dioxide reduction reaction (CO_2_RR) catalyst Cu_2_O.^5^ *Ex-situ* methods looking at morphological changes and dissolved substances in the electrolyte determined the conditions under which the surface was unstable, but to understand the corrosion process, AP-XPS was needed. Through measuring *operando* catalyst oxidation states in two electrolytes with AP-XPS, and we found that Cu_2_O undergoes both oxidation and reduction while under illumination. The oxidative process that occurs in aqueous electrolytes forms electron traps that prevent generated photoelectrons from catalyzing CO_2_ reduction reactions. This finding, combined with DFT calculations, suggested a protection scheme replacing the aqueous electrolyte with a nonaqueous one and adding a catalyst to speed transport of photoelectrons away from the Cu_2_O surface.^5^ In another work, we elucidated the self-improving nature of a GaN hydrogen evolution reaction (HER) photoelectrochemical catalyst using the *operando* technique photoconductive AFM (pc-AFM) along with *ex-situ* electron microscopy. By measuring the photocurrent at different crystallographic facets of the GaN, we found significant increases in the photocurrent generated at the GaN side walls as compared to top facets. *Ex-situ* scanning transmission electron microscopy coupled with electron energy loss spectroscopy (STEM-EELS) identified the compound on the side walls as gallium oxynitride, formed by oxygen substitution at a fraction of the nitrogen sites. This understanding of the stabilizing force of GaN illuminates a path toward more durable and higher performing photoelectrodes.^6^ These examples highlight the unique power of *operando* characterization methods in revealing critical insights about reaction mechanisms and material transformations, uncovering phenomena overlooked by *ex-situ* characterization. These mechanistic insights directly guide the rational optimization of catalysts and photoelectrodes.

In this perspective, we aim to survey the most recent literature published since 2020, with a few landmark exceptions, to highlight limitations of existing measurement techniques, and describe how these techniques can be combined in a multimodal approach to gain a more complete understanding of changing catalytic systems under operation. We will focus on *operando* methods for quantifying morphology, chemical species, and functional changes at catalytic interfaces to better understand reactions important to solar fuels. For readers interested in detailed discussions of these individual methods, we point to the comprehensive virtual issue in *Chemical Reviews* on “*In-Situ* and *Operando* Methods for Catalyst Characterization,” which covers many of the techniques discussed in this perspective. These include methods based on various probes such as X-rays and neutrons (scattering),^7–10^ photons (IR, Raman, UV–vis spectroscopy),^11,12^ scanning probes,^13^ and electrons (microscopy).^14^

Several key reactions in solar fuel research include the hydrogen evolution reaction (HER), oxygen evolution reaction (OER), CO_2_ reduction reaction (CO_2_RR), and carbon monoxide reduction reaction (CORR). Additional relevant techniques come from research on fuel cells, including research on catalysts for the oxygen reduction reaction (ORR), and fuel oxidation catalysts, with the CO oxidation reaction often employed as a model system for studying vapor-phase catalysis. Battery research also heavily involves electrocatalysts and uses imaging and measurement methods relevant to this field, and some examples will be presented here. Because illumination is critical for understanding the behavior of some PEC and PC catalysts, (i.e., dark measurements do not replicate the same events) we will highlight a few specific techniques used to introduce light to the surface during measurement, and the challenges there are. As shown in Figure 1 and Table 1, the examples of illuminated *operando* measurements presented here are involving X-ray photoelectron spectroscopy (XPS), X-ray absorption spectroscopy (XAS), Fourier Transform InfraRed (FTIR), and scanning probe microscopy (SPM)-based methods, some of the most common methods used to study catalytic evolution under illumination.

**Table 1:**
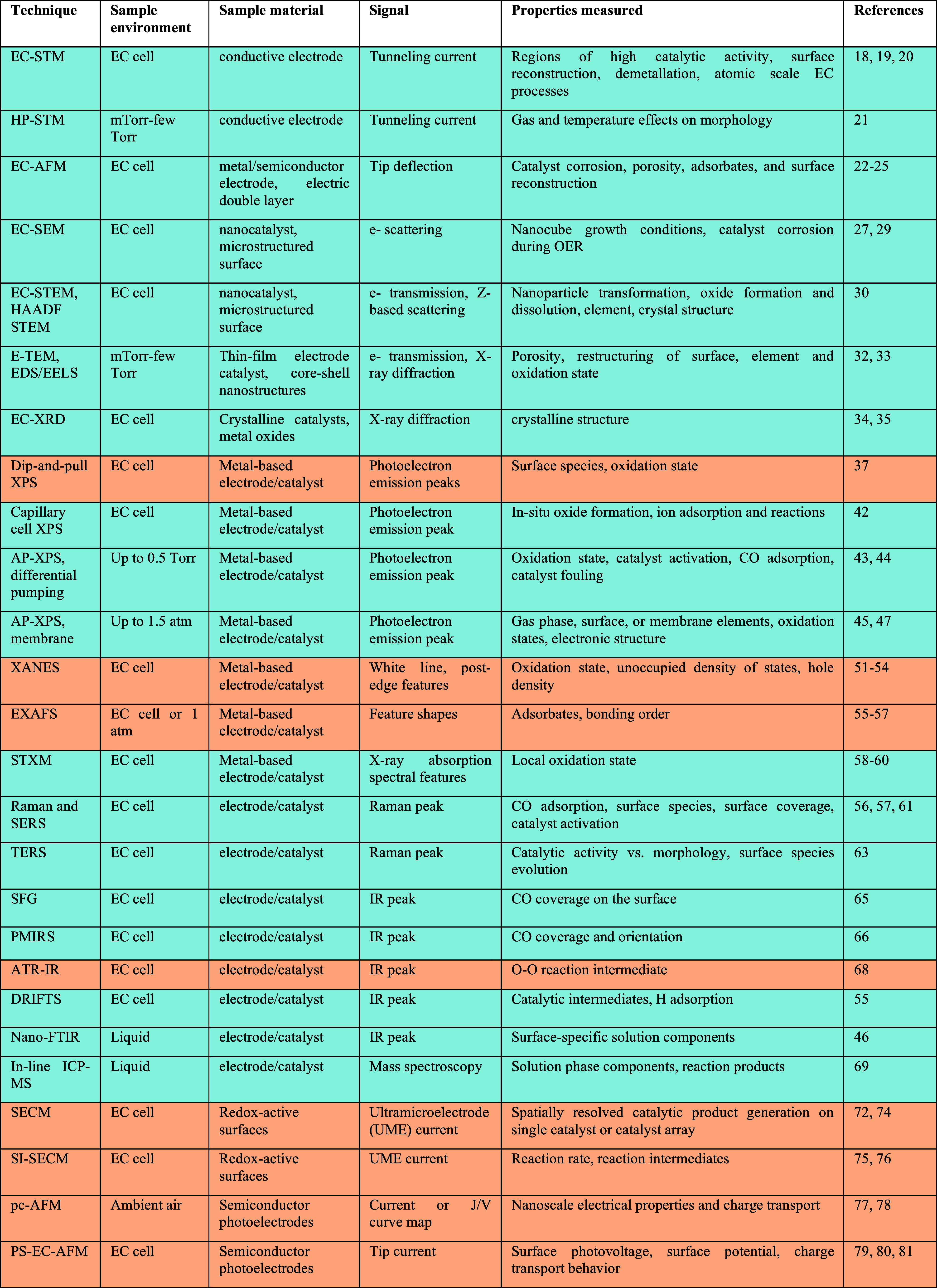
*Operando* Methods for Studying (Photo)­electrocatalysts^a^

a Blue rows indicate dark measurements, while orange rows indicate that at least one of the listed references provides an example of *operando* measurement setup under illumination.

Table 1 summarizes the methods discussed here using recent key examples from the literature, which represent either landmark developments or the most up-to-date findings available. We indicate whether the showcase studies concern metal or semiconductor materials and list representative compositions, thereby underscoring the versatility of each technique across distinct classes of catalytic and/or (photo)­electrode materials. Accordingly, the table specifies, for each *operando* technique, the compatible reaction environment, the recorded signal, and the physicochemical property probed, thereby offering a concise decision matrix for method selection in probing catalyst-electrolyte interface. Blue rows indicate dark measurements, while orange rows indicate that at least one of the listed references provides an example of *operando* measurement setup under illumination.

In the following sections, we will conduct an in-depth examination of *operando* methodologies, analyzing their specific operating conditions, key insights, and constraints. A systematic evaluation of these methods will elucidate both their analytical capabilities and inherent limitations.

Evolving catalyst surface morphology plays a crucial role in determining catalytic activity, selectivity, and stability. These dynamic structural transformations can include surface reconstruction, facet evolution, particle aggregation, and the formation of new phases, all of which significantly impact the catalyst’s performance. High resolution *operando* images of morphological changes down to the nanoscale are accessible by *operando* scanning probe and electron microscopy methods, namely: electrochemical scanning tunneling microscopy (EC-STM), atomic force microscopy (EC-AFM), scanning electron microscopy (EC-SEM), scanning transmission electron microscopy (EC-STEM); and outside the electrochemical cell with high pressure scanning tunneling microscopy (HP-STM) and environmental transmission electron microscopy (E-TEM), as well as X-ray diffraction methods.

While scanning probe and electron microscopy methods both enable *operando* observation of surface dynamics, they differ fundamentally in their operational mechanisms and capabilities. EC-SPM techniques function in a liquid electrolyte environment at ambient pressure, achieving atomic-scale resolution in *operando* measurements (0.1–1 Å vertical, 1–2 Å lateral for EC-STM^15^) through precise probe-surface interactions, enabling monitoring of atomic and molecular processes at the electrode–electrolyte interface. When sample illumination is required, a specialized cell allowing for simultaneous illumination and electrochemical measurements is needed (see the discussion on kinetics and functional properties later for some examples). This configuration enables direct observation of light-driven interfacial processes under realistic conditions, though careful consideration must be given to achieving uniform light distribution and accurate quantification of photon flux at the nanoscale.^16^ SPM cells would also be of use for tracking processes like light-driven corrosion.

While SPM techniques excel at capturing local surface phenomena such as atomic step evolution, surface reconstruction, and adsorbate dynamics in relatively simple *operando* setups, they often suffer from small field of view and slow imaging speeds. In contrast, electron microscopy techniques operate under restrictive environments, either at low pressure for E-TEM (1–20 Torr), or liquid-cell EC-TEM/SEM techniques that confine the sample between electron-transparent membranes with nm- to μm-thick liquid layers, while maintaining high vacuum in the remainder of the electron path. While being more restrictive in experimental conditions, electron microscopy compensates with broader field of view capabilities and faster image acquisition compared to SPM, while providing nm to μm resolution. This makes EM particularly valuable for observing larger-scale phenomena such as particle growth, dissolution processes, and morphological changes across extended surface areas. Additionally, electron microscopy techniques offer unique capabilities in elemental analysis and crystal structure determination through electron diffraction. One challenge for using EM with PEC and PC measurements is that illumination can present challenges because of the potential interference by the electron beam with sample excitation by illumination. However, there have been recent advances in specialized holders allowing illuminated TEM.^17^

SPM collects information by scanning a sharp tip across the surface in a raster pattern. Scanning Tunneling Microscopy (STM) and Atomic Force Microscopy (AFM) are two common methods used to measure surface morphology. In general, SPM can be adapted with specialized electrochemical cells to *operando* conditions, because the techniques themselves do not tend to require high vacuum for functionality. Besides STM and AFM, additional methods exist to measure other surface properties including chemical and electrochemical properties, discussed below.

Recent technological advances have significantly enhanced AFM scanning speeds for some microscopes, evolving from traditional time-consuming measurements (5–10 min per moderate resolution scan, or hours for high-resolution/large-area imaging) to achieving scan rates of just a few seconds, enabling dynamic studies of rapid morphological changes. Temporal resolution can be improved by strategic measurement approaches, such as combining initial surface scans with targeted I/V or force curve measurements at specific locations during catalysis. However, several practical considerations remain crucial for reliable EC-AFM measurements: fluid dynamics can significantly affect tip motions and resonant frequencies, while setup and stabilization in a fluid cell often requires careful optimization. Additional challenges include sample and tip contamination due to direct tip–sample interaction, maintaining uniform tip parameters to avoid imaging artifacts, and managing interference from particles or evolved bubbles. These limitations become particularly significant when studying rapidly degrading samples, as the sequential nature of AFM scanning means different parts of an image are captured at different points in the catalyst’s lifetime, potentially complicating data interpretation.

In STM, surface imaging is carried out by measuring tunneling current between a conductive surface and a sharp tip. STM is often done under high vacuum to ensure the surface is free of contamination, but electrochemical STM (EC-STM) extends its capabilities to function in electrolyte solutions, enabling *operando* imaging of electrode surfaces with atomic resolution. While EC-STM implementation initially faced significant challenges with faradaic current interference and mechanical stability issues in liquid environments, these obstacles have been overcome, making it a reliable tool for high resolution imaging under electrochemical conditions. Some advances that were particularly valuable were the development of sophisticated tip coating protocols and optimized imaging cells.^18^ In addition, recent advances in digital signal processing, novel coating materials, and integrated spectroscopic capabilities have further expanded EC-STM’s potential for studying dynamic electrochemical processes with unprecedented spatial and temporal resolution. A recently developed current roughness (cr-EC-STM) approach quantified local catalytic activity from tunneling-current fluctuations and was applied to study the HER at graphene-metal interfaces. Noise analysis revealed that single iron atoms in carbon vacancies and curved graphene regions exhibit exceptional catalytic activity, even exceeding that of platinum. The cr-EC-STM results correlated with macroscopic HER trends, underscoring the role of point defects and demonstrating the technique’s power for high-resolution electrocatalytic mapping.^19^ Complementary advances in tip-coating materials have further enhanced EC-STM performance. Notably, even a simple nail polish coating has been shown to suppress faradaic currents under electrochemical conditions, enabling the direct observation of structural changes in an FePc ORR catalyst.^20^ Beside EC-STM, another STM method used in *operando* imaging is high pressure STM (HP-STM), where a fixed low pressure (mTorr-few Torr) of gases is introduced into the STM chamber to study their interaction with catalysts for vapor phase reactions. This approach enables direct visualization of catalytic interfaces under realistic reaction conditions, providing crucial insight into surface dynamics relevant to fuel-cell and oxidation reactions. For example, HP-STM has mapped site-dependent activity on Co/CoOx catalysts and captured their transformation into nanoparticles, correlating with enhanced catalytic performance due to increased surface area and favorable oxidation-state changes.^21^ These examples collectively demonstrate that advanced STM techniques provide critical insights into the dynamic restructuring, defect engineering, and intermediate formation at catalytic interfaces, offering a foundation for optimizing solar fuel catalysts through enhanced understanding of surface reactivity and structural evolution under *operando* conditions.

While STM relies on tunneling current for imaging, AFM detects deflection of a sharp tip from the sample surface to map surface topography. Due to its relatively simple setup and robust operation, AFM is readily done in an electrochemical cell, or under controlled temperature, pressure, humidity, illumination, etc., making it simple to design *operando* measurements of catalytic systems. Although electrochemical AFM (EC-AFM) typically provides lower spatial resolution compared to EC-STM, its ability to operate independently of sample conductivity and integration with new measurement methods makes it applicable to a broader range of materials including semiconductors, insulators, and soft electrodes materials. Taking advantages of these capabilities, EC-AFM can effectively measure changing surface morphology of an electrode during an electrochemical process, enabling direct observation of physical changes like corrosion, adsorption, or remodeling. For example, we gained insight into the mechanism of BiVO_4_ photoanodes’ degradation process using EC-AFM, revealing the progressive dissolution of both BiVO_4_ grain top surfaces and side walls (Figure 2a,b). The EC-AFM data helped elucidate how nonequilibrium and kinetic factors influence the stability of the material beyond what thermodynamic predictions would suggest.^22^ EC-AFM can also lend insight into structural dynamics. *Operando* EC-AFM equipped with photothermal excitation of the cantilever oscillation has enabled high-resolution imaging of catalytic surfaces in electrolyte by minimizing thermal noise and enhancing imaging speed in electrolyte during electrochemical reactions. This capability allowed nanoscale visualization of potential-dependent surface restructuring and adsorbate formation during CO_2_RR, revealing significant morphological changes between the reaction onset and the potentials leading to C_2_^+^ products (Figure 2c,d).^23^ Further advances in EC-AFM instrumentation have pushed spatial resolution to the atomic scale under electrochemical conditions. Frequency-modulation EC-AFM, combined with conductive tip coatings, has achieved atomic-lattice-resolution imaging under electrochemical conditions by overcoming corrosion and stability challenges in liquid environments. This approach enabled direct visualization of interfacial water dissociation on oxide surfaces within the potential range relevant to the OER.^24^ While conventional EC-AFM provides valuable insights into surface morphology, the development of charge profiling 3D AFM, a combination of EC-AFM with precise statistical and other data analysis, represents a significant advancement by enabling three-dimensional mapping of the electrode–electrolyte interface through controlled probe positioning in both lateral and vertical directions. This technique goes beyond traditional surface imaging by providing detailed volumetric maps of the electrical double layer (EDL), revealing Angstrom-scale precision variations in ion distribution and solvation shells, calculated by systematic force mapping. The ability to monitor EDL structure changes in response to varying electrochemical potentials makes 3D-EC-AFM particularly valuable for understanding molecular-level processes at catalytic interfaces, helping bridge the gap between theoretical models and experimental observations of reactant and product behavior.^25^

**Figure 2:**
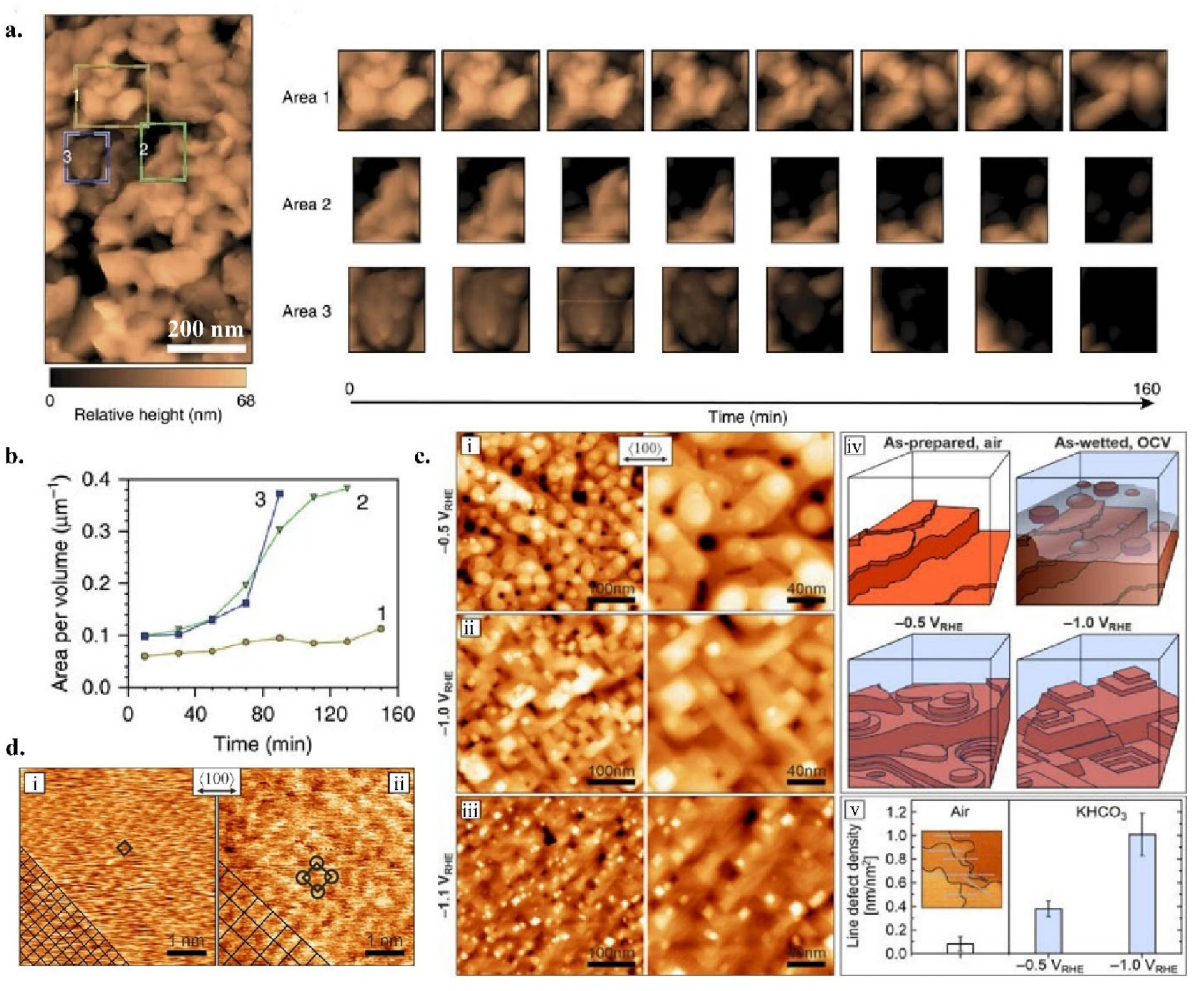
(a) EC-AFM scan of BiVO_4_ working electrode surface to examine the degradation process over a period of 160 min. Three colored boxes indicate Regions 1 (yellow), 2 (green) and 3 (blue), whose temporal evolution were tracked in detail. (b) Progression of the surface area to volume ratio for the three regions over the course of the 160 min test. Panels a and b are reproduced from ref 22. Available under license CC-BY-4.0. Copyright 2016 the authors. (c) Surface morphology of Cu(100) under different applied potentials during CO_2_RR. (i–iii) *In-situ* EC-AFM images of electropolished Cu(100) in CO_2_-saturated 0.1 m KHCO_3_ recorded at −0.5, −1.0, −1.1 VRHE. (iv) Cross-section schematics of morphologies observed in this study. (v) Bar diagram of the line-defect densities extracted from *operando* AFM images at the respective surface conditions. (d) Atomic resolution *operando* AFM images of electropolished Cu(100) surfaces under different reducing potentials in CO_2_-saturated 0.1 m KHCO_3_. (i) At −1.0 VRHE a (1 × 1) surface is present. (ii) At −0.5 VRHE a p(2 × 2) superstructure has been observed. Unit cells are indicated by black squares. The double arrow marks the nominal bulk copper <100> direction for both frames, actual alignment is indicated by the square lattices in the bottom left corners. Panels c and d are reproduced from ref 23. Available under license CC-BY-4.0. Copyright 2021 the authors.

Electron microscopy (EM) utilizes electron beam interactions with the electron cloud of a sample to generate images with resolution that far surpasses light microscopy (due to the small wavelength of electrons). However, because electrons have significant mean free paths only under high vacuum, *operando* EM has fundamental limitations to contend with. Two basic approaches are taken to address this. In one, the sample environment is encapsulated in a thin cell with gas and/or liquid layers over the sample and a graphene or SiN cell window. In the other, differential pumping is used to maintain a high vacuum at the analyzer and much of the electron path, with a low pressure of gases over the sample.^26^ In a liquid cell, the resolution attainable at the solid/liquid interface is limited by the thickness of the liquid layer the beam passes through, due to electron scattering off the liquid molecules. A recent meta-analysis of *operando* electron microscopy showed that while a liquid thickness of 100 nm provides resolution of around 0.1 nm, a 10 μm layer of liquid limits the resolution to around 10 nm.^26^ Therefore, the depth of the liquid layer is minimized as much as possible while still maintaining *operando* conditions. Additional background on *operando* EM can be found in recent reviews including Chee et al.^14^

SEM is a fast, high-resolution method to image a conductive surface by scanning an electron beam in a raster pattern across the surface and measuring the energy of electrons reflected or scattered off the surface. SEM scans are often faster than scanning probe measurements, since the beam is controlled by optics rather than physical sample/tip interactions. Unlike scanning probe methods, SEM can also image surfaces with large features and/or high roughness accurately, because there is no physical tip that must track the surface. *Operando* SEM is often done using a thin cell and true electrochemical conditions to study catalytic reactions. In addition to the challenges posed by setting up the sample in the specialized EC-SEM cell, SEM is not feasible on nonconductive surfaces, posing a limitation on the types of samples that can be imaged. The development of specialized electrochemical cells for SEM has enabled real-time observation of morphological evolution during catalytic processes across multiple length scales, from individual nanoparticles to extended surface features.^27^ A recent advancement in *operando* EC-SEM introduced a microcell based on a free-standing, trilayer graphene sheet serving simultaneously as the working electrode and electron-transparent viewing membrane. Integration with external reference and counter electrodes simplified the design and improved spatial resolution compared to conventional SiNx-based cells. The configuration also provided a wider inert potential window than glassy carbon, enabling *operando* monitoring of Cu nanocube degradation at realistic CO_2_ electroreduction potentials. This work provides a powerful example of how novel material integration in microcell engineering can push the boundaries of observing electrocatalytic processes under realistic operating conditions.^28^ Beside nanoparticles, EC-SEM has also enabled real-time visualization of morphological and structural evolution in RuO_*x*_ catalysts surface during the OER, capturing oxidation, deprotonation, and dissolution leading to the transition from a microstructured to a smoother surface (Figure 3a,b). Additionally, EC-SEM highlighted the stability issues associated with amorphous RuO_*x*_, illustrating the trade-off between higher activity and lower stability. Overall, it complemented the electrochemical data, enhancing the understanding of the relationship between film morphology, electrochemical performance, and catalytic mechanisms.^29^

**Figure 3:**
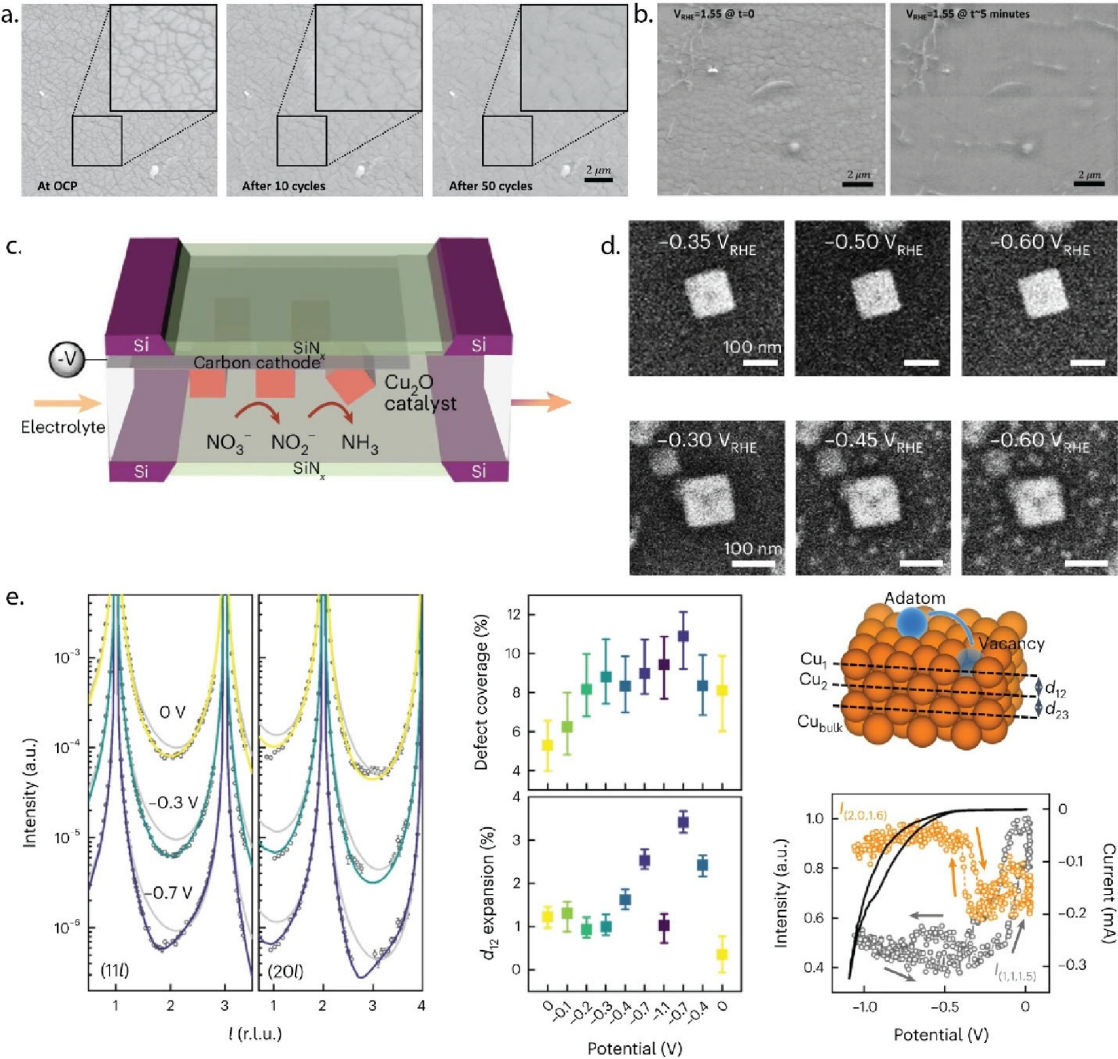
(a) *Operando* EC-SEM micrographs of a ruthenium film covered with graphene at open circuit potential, after 10 cycles, and after 50 cycles of anodic polarization. (b) Dissolution of ruthenium oxide at anodic potentials 1.55 V_RHE_ after approximately 5 min. Panels a and b are reproduced from ref 29 Available under license CC-BY-4.0. Copyright 2023 the authors (c) Schematic of the EC-TEM setup, where Cu_2_O precatalyst is electrodeposited on the working electrode of the chip. (d) *Operando* EC-TEM snapshots track Cu_2_O cube restructuring during linear sweep voltammetry in nitrate reduction reaction electrolyte (top) and, at similar potentials, in CO_2_RR electrolyte (bottom). Panels c and d are reproduced from ref 31. Available under a Creative Commons Attribution 4.0 International License. Copyright 2025 the authors. (e) *Operando* SXRD reveals potential-induced restructuring of Cu(100) at 0, −0.3 and −0.7 V (circles) match fits (solid lines) to a model with adatom-vacancy pairs and relaxed d_12_/d_23_ spacings, diverging from ideal, defect-free surfaces (gray). Fits quantify a cathodic increase in adatom-vacancy coverage and d_12_ expansion, while real-time intensities at (2 0 1.6) and (1 1 1.5) monitor structural evolution during CV scans. Panel e is reproduced from ref 35. Available under a Creative Commons Attribution 4.0 International License. Copyright 2023 the authors.

TEM is a method to study crystal structure and morphology with electrons scattered by or transmitted through a thin sample that is placed in an electron beam. It is often combined with the electron diffraction method high-angle annular dark-field (HAADF) to create a map with atomic number contrast in the sample. X-rays emitted from the sample during the measurement can give additional information on elemental analysis using energy dispersive X-ray spectroscopy (EDX), and material thickness, electronic states, and bonding can be determined through measuring the energy distribution of the transmitted electrons in Electron Energy Loss Spectroscopy (EELS). From these techniques, TEM can provide high resolution maps of the chemical species on the catalyst surface as well as morphology.

Electrochemical TEM (EC-TEM) methods rely on similar thin cell setups as EC-SEM, and it can be used to study real-time structural changes such as sintering or nanoparticle nucleation and growth. One common EC-TEM method is Scanning Transmission Electron Microscopy, or STEM, which scans an electron beam as in SEM, but measures transmitted electrons through the sample rather than those scattered off the surface. *Operando* EC-STEM, combining high spatial resolution with real-time imaging, has advanced the visualization of catalyst restructuring during CO_2_ reduction. Spatial resolution is largely determined by the electrolyte thickness, which can be reduced to ∼ 100 nm to enhance imaging quality. 4D-STEM acquisition enables time-resolved diffraction, providing atomic-scale insight into nanocatalyst transformations and making this technique suited to track structural dynamics and guide future catalyst design.^30^ Paired with *operando* X-ray and Raman spectroscopies, a recent work employs EC liquid-cell TEM to directly visualize and correlate the potential-dependent transformation of Cu_2_O nanocubes during nitrate reduction, uncovering a kinetically stabilized Cu_2_O/metallic-Cu coexistence that governs ammonia selectivity (Figure 3c,d).^31^

In some cases, to observe *operando* interaction of a catalyst with gases, environmental TEM (E-TEM) is used, where differential pumping is applied to maintain a controlled low pressure (mTorr - few Torr) of gases over the sample. Specifically, this method recently enabled to visualize structural changes, catalyst activation, and fuel cell operation in a solid oxide fuel cell. This approach included sample heating to a maximum of 750 °C under ∼10 Torr of H_2_/N_2_ or H_2_/N_2_/O_2_.^32^ By operating under realistic reaction conditions, E-TEM provided strong evidence of the kinetically trapped active phase, bridging gaps between theory and *operando* performance.^33^

Moving beyond the surface topography revealed by EC-SPM and EC-EM, *operando* X-ray diffraction (XRD) provides quantitative, real-time analysis of the catalyst’s underlying crystallographic structure during electrochemical operation. The technique’s principle lies in monitoring the diffraction pattern from a working electrode/catalyst to track changes in crystal structure, lattice parameters, and surface morphology, thereby enabling a direct correlation between atomic-scale transformations and applied electrochemical stimuli. Historically, *operando* XRD studies were hindered by significant limitations, including low signal-to-noise from surface layers, poor temporal resolution, and interference from the complex electrochemical environment. The advent of high-brilliance synchrotron light sources was a pivotal development, providing the immense X-ray flux needed to overcome the weak surface signal. This was complemented by the integration of high-speed 2D area detectors, which drastically reduced acquisition times, and the engineering of sophisticated flow cells that mitigate interference from gas evolution and electrolyte absorption. Collectively, these advancements have transformed the technique, enabling the real-time tracking of atomic-scale catalyst evolution with high temporal and structural precision. *Operando* high-energy surface XRD has proven uniquely robust, resolving the highly transient oxide that reveals Pt(100) degradation during electro-oxidation^34^ and capturing the rapid restructuring of Cu(100) under gas-evolving CO_2_RR conditions where other high-resolution probes like STM often fail.^35^ These works highlight the maturation of *operando* XRD by pushing the limits of temporal resolution to capture fleeting intermediates, and extending the technique’s environmental resilience to probe catalysts under challenging, industrially relevant conditions.

While morphology sets the stage for catalysis, the chemical species involved are the active participants driving the reaction, making their study indispensable for understanding and improving catalytic performance. During catalysis, changes in catalyst oxidation state, bonding, adsorption/desorption, and surface reactions are paramount because they directly elucidate the atomic and molecular processes that define catalytic mechanisms. To track these dynamic changes, various *operando* spectroscopic methods have been developed, each offering unique capabilities. To quantify oxidation state and chemical species changes during an catalytic reaction, ambient pressure X-ray spectroscopy (X-ray photoelectron spectroscopy and X-ray absorption spectroscopy) and vibrational spectroscopy (Raman and Fourier-transform InfraRed or FTIR) methods quantify chemical species largely without spatial resolution, but the recently developed techniques Tip-enhanced Raman Spectroscopy (TERS) and nano-FTIR methods extend Raman and IR methods to create spatial resolution by visualizing surface chemical species at the point of tip–sample contact. As discussed in the previous section, the TEM based methods high-angle annular dark-field imaging (HAADF), Energy Dispersive X-ray Spectroscopy (EDS) and Electron energy loss spectroscopy (EELS), also correlate topography with chemical species.

XAS and XPS excel at probing electronic structure, bonding, and oxidation states of catalysts, while Raman and FTIR spectroscopy reveal vibrational modes that indicate molecular structure and surface species formation, as well as identifying adsorbed intermediates and monitoring reaction processes through characteristic molecular vibrations. Complementing these techniques, in-line mass spectroscopy enables real-time detection and quantification of reaction products. There exist some technical challenges to X-ray spectroscopy in an electrochemical cell, because similar to in electron microscopy, the instrument requires high vacuum. On the other hand, FTIR must deal with strong interference from electron absorption, and FTIR and Raman both lack surface selectivity. These methods’ challenges have driven the development of specialized cells and measurement configurations to bridge the “pressure gap” between traditional surface science and realistic catalytic conditions.

The integration of illumination sources presents additional challenges in spectroscopic techniques. However, novel synchrotron-based ambient-pressure XPS configurations enable *operando* measurements while maintaining compatibility with UV/visible illumination sources. XAS capabilities under illumination have also been enhanced through modeling and specialized cell designs that better account for light scattering effects in liquid media, though probe depth limitations remain a fundamental constraint (1–3 μm in the gas phase, <1 mm in the liquid phase).^36^ The development of illumination systems for Raman spectroscopy in photocatalysis has progressed significantly through the integration of AM1.5-compatible light sources and fiber optic delivery systems, enabling real-time monitoring of structural and chemical changes while maintaining spectroscopic sensitivity. The illumination can create interference with Raman signal collection and expose the sample to excessive heat, necessitating careful optimization of experimental parameters.^37^

XPS assesses elemental composition and oxidation state of a catalyst surface. While oxidation state and surface composition are often key factors of catalyst operation, *operando* XPS is extremely valuable to track their changes. In XPS, the sample is irradiated by X-rays while the detector measures the energies of photoelectrons emitted from core orbitals of surface atoms. Because photoelectrons are absorbed by interaction with atoms, XPS is most sensitive for measurements in high vacuum, and in many setups the X-rays do not penetrate more than a few nm into the sample, making it a surface-sensitive technique. To address this issue, several methods to perform *operando* ambient pressure XPS (AP-XPS) have been developed and are widely used. First, synchrotron sources are often used for AP-XPS, because they can generate higher energy and intensity X-rays needed to penetrate vapor/liquid layers, and generate high kinetic energy photoelectrons that will reach the detector. Second, to create an *operando* environment for the sample, either a sealed sample cell or differential pumping is used to separate the detector from the gas/electrochemical cell.^38^ For photodependent processes, a solar simulator can be incorporated to provide illumination to the sample during measurement. Challenges of *operando* XPS include the need for a synchrotron X-ray source, as well as the extensive setup, calibration, and testing time required. Finally, the signal is often low magnitude and noisy because of the strong attenuation by the liquid and vapor phase of emitted electrons from the sample before they reach the detector, as well as interference from solution phase and subsurface layers. This issue gives a degree of uncertainty to extracted peak intensities. Despite these limitations, it provides invaluable information about the transformation of the surface during catalysis.

In many configurations, electrochemical AP-XPS uses a very thin layer of electrolyte in the beam path, with additional electrolyte in an adjacent beaker or droplet. A small pressure of water vapor via differential pumping is maintained over the liquid to prevent evaporation. A thin, stable liquid layer is needed to both decrease photoelectron reabsorption and limit X-ray damage to the electrolyte components. One common technique is the dip-and-pull method, in which the catalyst is pulled out of a beaker of electrolyte, leaving a thin film of solution clinging to the surface (Figure 4a). The solid surface is analyzed through this thin liquid film while electrochemistry is done on the submerged catalyst. In a recent study from our group, we used dip-and-pull AP-XPS in a synchrotron beamline along with a solar simulator to study unstable corrosion intermediates generated by the photoelectrochemical CO_2_RR catalyst Cu_2_O. Analysis of the O and Cu peaks from the vapor, liquid, and the solid phases shed light on the rate of oxidation and corrosion of Cu_2_O in different electrolytes (Figure 4b,c).^5^ The dip-and-pull method relies on a differential pumping technique and a thin 20 nm film of electrolyte, which allows the surface to be probed under precatalytic and catalytic conditions. Active and inactive forms of the catalyst can be quantified by peak deconvolution, thus providing information on the active species during the reaction.^39^ On the other hand, the dip-and-pull method remains limited by the requirement of an ultrathin (few-nanometer) electrolyte layer, high Ohmic drop, and severe mass-transport constraints, making realistic reaction conditions difficult to achieve. Addressing this gap, Davies et al. recently introduced a significantly modified setup that integrates hard X-rays with a thicker electrolyte, a dedicated gas nozzle to overcome diffusion bottlenecks, and a novel in-line electrode geometry that minimizes Ohmic drop to ensure accurate potential control. This combination of innovations overcomes the primary obstacles for studying gas-consuming electrocatalysis, providing a new window into surface-based reaction mechanisms under operating conditions.^40^ A complementary thin layer electrolyte method was further developed by using a specially designed miniature capillary device to create a two- or three-electrode electrochemical cell in a thin-layer configuration. The cell is placed close to the analyzer, allowing for smaller samples. The design reduces the Ohmic drop due to the proximity of reference and counter electrodes to the working electrode. It can be integrated into most AP-XPS systems as a bolt-on device.^41,42^

**Figure 4:**
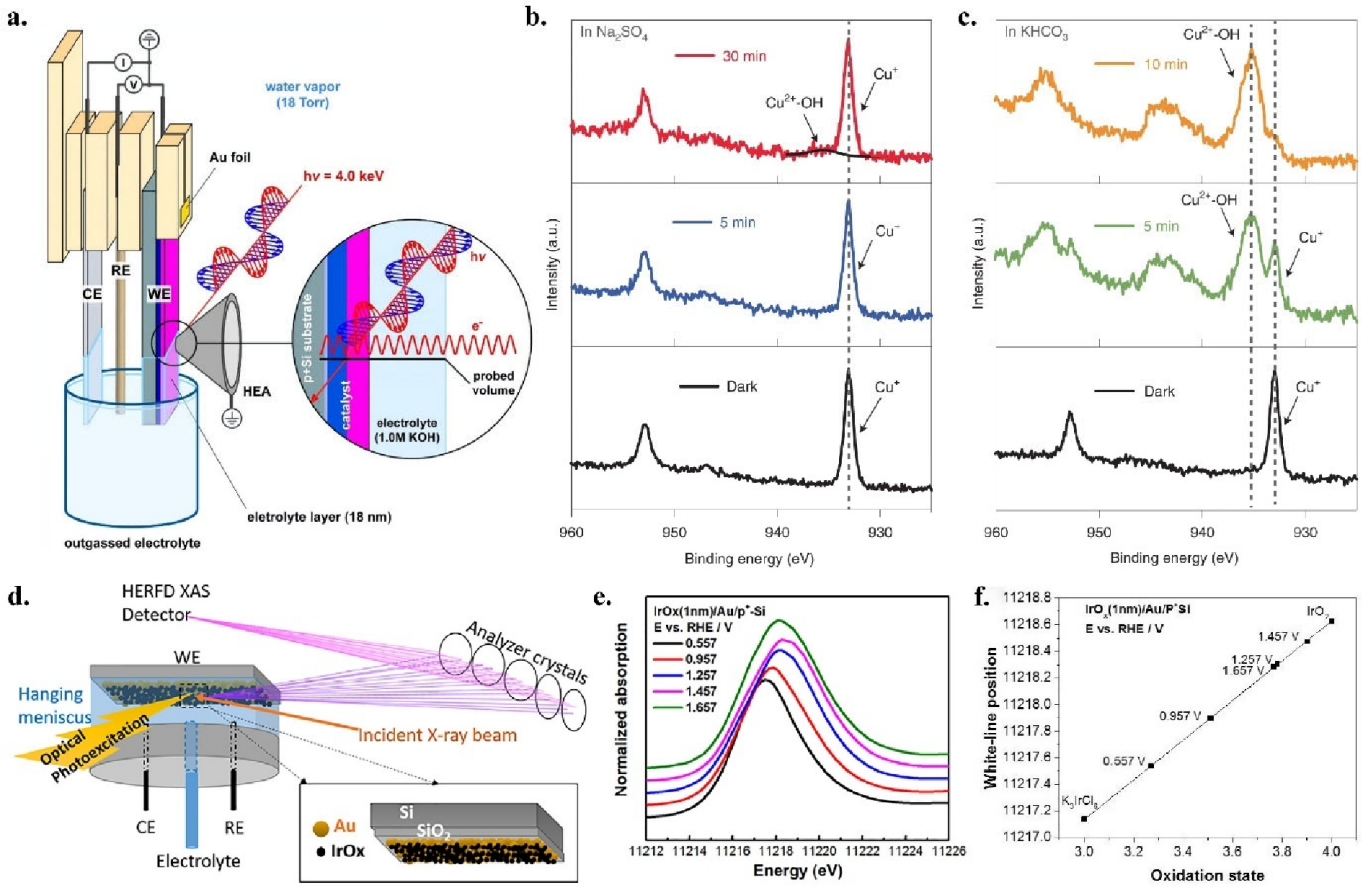
(a) Schematic of experimental setup. Panel a is reproduced from ref 39. Copyright © 2017 American Chemical Society. (b, c) Development of Cu^2+^-OH soluble product in Cu_2_O under illumination in KHCO_3_ (c) compared to Na_2_SO_4_ (b) electrolyte. Panels b and c are reproduced with permission from ref 5. Copyright © 2021, the authors, under exclusive license to Springer Nature Limited. (d) *operando* XAS setup using a hanging meniscus 3-electrode cell. (e) Ir white line position at potentials under and over OER onset. (f) using known oxidation states of Ir­(III) and Ir­(IV) compounds to map oxidation state of each surface in e. Panels d–f are reproduced with permission from ref 51. Copyright © 2019 American Chemical Society.

Besides electrochemical studies, *operando* AP-XPS *via* differential pumping alone is valuable to study catalyst interactions with vapor phase components. Pressures are commonly in the 100s of Torr. These pressures allow for studies of reactions in gas-phase, and for monitoring the dynamic evolution of surface chemistry under relevant reaction conditions. These chemical insights can be combined with E-TEM’s structural observations, as presented in the previous section, and provide a comprehensive understanding of how kinetically trapped metastable states govern catalytic performance.^33^ With the possibility to use the pressure range of 150–500 mTorr as well as high temperatures (∼550 °C), these examples highlight the unique capability of AP-XPS to bridge the gap between *ex-situ* surface chemistry and *operando* catalytic behavior, offering critical guidance for designing and optimizing catalysts for selective oxidation processes.^43,44^

In some cases, gas pressures of 1 atm or greater are needed to assess realistic gas interactions with the surface. For these measurements, the sample is separated from the detector using a graphene or metal oxide membrane balancing transparency to photoelectrons with mechanical strength and impermeability to gas and liquid.^45,46^ Specifically, graphene has been utilized with several catalysts such as Cu,^45^ Ir, and Pd on graphene membranes, tracking oxidation state changes and catalytic reactions like propyne hydrogenation on Pd/C.^47^ However, graphene’s photoemission peaks can interfere with carbon-based samples. To address this limitation, thin TiO_2_ or Al_2_O_3_ membranes have also been developed, allowing AP-XPS measurements of 1 atm air with high photoelectron transparency.^46^

XAS is another powerful tool for *operando* catalytic interface studies, as it probes the local electronic structure and atomic coordination of active sites during reaction conditions. Unlike XPS, which largely identifies elements and their oxidation state, in XAS high energy X-rays excite electrons from core orbitals to higher states. The core hole is later filled by another electron, emitting a photon or secondary electron in the process. Both emitted electrons (Total Electron Yield/TEY or Auger Yield/AY) and photons (Fluorescence Yield/FY) are associated with the outer shell electron filling the core hole, providing information about the energy levels of unoccupied states. Challenges of XAS include requiring a synchrotron source for the measurement and attenuation of emitted electrons by the electrolyte, affecting sensitivity. This has led to a variety of sample procedures and cell designs, including those that accommodate an illumination source. Another challenge with XAS is that unlike XPS, it is not inherently a surface selective technique, so can have interference from bulk states in the sample. High energy resolution fluorescence detection XAS (HERFD-XAS), which isolates an ∼1 eV slice of a chosen fluorescence line, achieves significantly sharper absorption spectra while effectively suppressing the electrolyte’s dominant elastic and Compton scattering background. Recently, *operando* soft X-ray TXM and scanning transmission X-ray microscopy (STXM) were developed that enable site-specific analysis of chemical and structural changes within individual particles, revealing inhomogeneities and local reaction mechanisms that are hidden in bulk measurements. XAS is classified by which core level is probed, with K-, L-, or M- edge corresponding to excitation of electrons from the 1s, 2s/p, or 3s/p/d orbitals, respectively. XAS is separated by the energy range it covers relative to the element’s absorbance edge. Below the absorbance edge, X-ray absorption near-edge structure (XANES) provides information about oxidation states and adsorbates. At energies above the absorbance edge, extended X-ray absorption fine structure (EXAFS) can reveal details about bond distances and coordination numbers of neighboring atoms.^48,49^

XANES measures energies of unoccupied, loosely bound surface states as well as chemical bonding and surface adsorbates. Bonding and oxidation state of the atom affects the position and height of the absorption edge and the shape of pre-edge features. Either modeling and simulation or reference spectra are used to extract oxidation and bonding states from the data.^44^ For example, we used XANES L-edge measurements to track average oxidation states in an *operando* IrOx/Au/p^+^n-Si OER photoanode. For *operando* electrochemical XAS measurements, a method frequently used is a ‘hanging meniscus’, which separates the electrochemical cell from the remainder of the chamber with a membrane. In our work, the hanging meniscus method was coupled with solar illumination and HERFD-XAS to probe photoanode behavior under realistic operating conditions (Figure 4d). Using FY detection, we tracked potential-dependent oxidation of IrOx layers of varying thickness, revealing greater oxidation for thinner films and identifying surface Ir sites as the dominant active species (Figure 4e, f).^51^ In another study, surface sensitivity in XANES was enhanced both by modulating X-ray photon energy and by using TEY detection rather than FY detection. Realistic gas pressure and elevated temperature reaction conditions were maintained around the electrode using a cell with a 100 nm SiN window to measure SnO_2_ nanoparticle surface states.^52^ Because of the challenge of electrolyte interference with the signal, techniques have been designed where the catalyst is deposited on a permeable substrate and an ion exchange membrane keeps the catalyst electrochemically connected to the cell without need for an electrolyte over it.^53^ A similar ion exchange membrane setup was used in conjunction with in-line GC to quantify catalytic products while simultaneously measuring oxidation states.^54^

EXAFS looks at XA characteristics above the absorption edge. This region is dependent on scattering, which gives it the benefit of being precisely mathematically modeled. A background level of absorbance is seen superimposed on oscillations resulting from wave interference between the emitted core electrons and the scattered electrons from neighboring atoms. By using mathematical modeling, the distances to, identity of and coordination number of neighboring atoms can be determined.^50^ EXAFS provides information on morphological changes at catalytic sites as well as providing chemical identification. The power of EXAFS to resolve local atomic environments is exemplified in the work of Lu et al., who combined *operando* EXAFS and XANES (Pt L-edge and O K-edge fluorescence yield) to decode the structure–activity relationship in a dual-site TiO_2_/Pt HER catalyst. XANES and EXAFS spectra collectively uncovered dynamic structural changes during reaction. These techniques not only mapped the static coordination environment but also captured the transient structural motifs driving catalysis, bridging atomic-scale insights with macroscopic performance.^55^ In addition, *operando* EXAFS has been pivotal in uncovering dynamic structural changes in catalysts, and in resolving dynamic transformations, alloy evolution, surface segregation, and molecular integrity.^56,57^

Beside bulk measurement, the application of *operando* TXM/STXM in electrocatalysis has advanced significantly over the past few years, moving from direct observation to sophisticated, multimodal analysis. The study by Mefford et al. established the technique’s power in creating nanoscale chemical maps to forge a direct link between the local oxidation state of a catalyst (Co^3+^) and its electrochemical activity.^58^ Building on this, Zhang et al. demonstrated a refined application using a custom microfluidic cell, which enabled the technique to not only track chemical changes but also to identify experimental artifacts by spatially distinguishing between catalytically active and electronically isolated nanoparticles, an insight impossible with bulk-averaging methods.^59^ The most recent work by Yoon et al. represents a further leap, integrating the technique into a correlated workflow where the same sample holder is used for both TEM and TXM. This multimodal approach, combined with other *operando* spectroscopies, provides a holistic view of catalyst restructuring, confirming that different chemical phases (Cu_2_O and metallic Cu) can coexist but remain spatially separate.^60^ This progression shows a clear trend toward using spectro-microscopy to resolve increasingly complex, heterogeneous catalyst behaviors and to build more complete and reliable structure–activity relationships by correlating data across multiple advanced characterization platforms.

The vibrational spectroscopy techniques Raman and FTIR spectroscopies are complementary techniques that provide information on the molecular stretching frequencies (FTIR) and dipole changes (Raman). Adsorbates and reaction intermediates can be identified under reaction conditions, though *operando* measurements require specialized cells to reduce signal attenuation and attain maximum sensitivity to the catalyst surface.

Raman spectroscopy is a powerful way to identify compounds immobilized on a surface by measuring their vibrational modes. Specific molecules have a ‘fingerprint’ of vibrational modes, which can be probed by looking at the energy shift of inelastically scattered light compared to the energy of the incident (usually visible light or near-visible) beam. Raman is readily done under ambient conditions, so it lends itself well to *operando* measurements, and it does not have interference from electrolytes as FTIR does. Raman is not inherently surface-specific, so signals from surface species may have weak Raman signals because of dilution by the bulk. Numerous methods have been designed to increase the surface signal relative to the bulk. *Operando* Raman spectroscopy allowed direct visualization of CO adsorption on a Cu cathode surface through tracking the Cu-CO and Fe-CO Raman peaks using porous PTFE as the cathodic catalyst support. Along with the XAS analysis mentioned in the last section, this suggested a mechanism for the observed product selectivity improvement, where CO_2_ is reduced to CO on the catalyst surface, thus increasing selectivity for EtOH production.^56^ *Operando* Raman approaches have also elucidated adsorption behavior in other catalytic systems. For example, Xu et al. observed an increased Ni–O peak of NiFe, as the catalyst became more activated correlated with improved catalytic activity.^61^

To enhance surface signals in Raman, Surface-Enhanced Raman Spectroscopy (SERS) uses a rough metal surface to increase the signal from the desired surface sites. The mechanism behind the enhancement is thought to be due to the positive relationship between the local electric field at the surface and the scattering intensity. In SERS, the sample is deposited on the rough metal surface, then illuminated with a laser, generating a localized surface plasmon resonance (LSPR) and enhancing Raman scattering by surface molecules. The LSPR arises from the confinement of the electron cloud in the conductive nanoscale features, in which is induced an oscillation by the resonant frequency of the incident light, generating a localized electric field stronger than that of the laser itself. The LSPR electric field enhances the intensity of both the incident light on the sample, and the scattered light from the sample. Rough surface features around 10 nm in diameter are often used in SERS. Like standard Raman measurements, SERS is compatible with electrochemical measurements with minimal modification (Figure 5a). Herzog et al. applied *operando* SERS (785 nm) to 35 nm Cu_2_O nanocube CO_2_RR catalysts, with and without 5 nm Ag nanoparticle decoration, to quantify adsorbed CO.^57^

**Figure 5:**
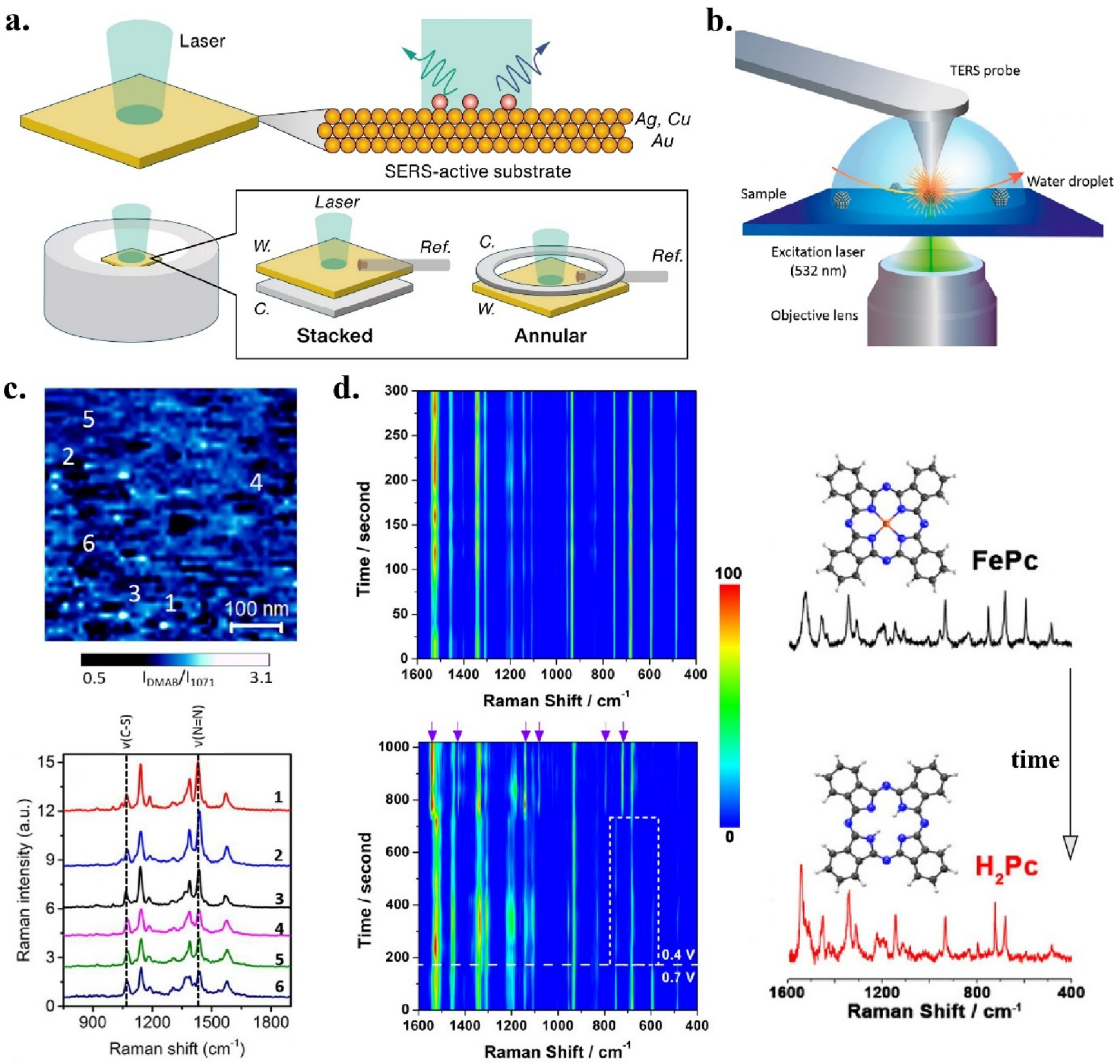
(a) Illustration of working electrodes as SERS-active substrate and two common electrode configurations for Raman spectroelectrochemical cell: stacked and annular arrangement. Figure 5a is reproduced with permission from ref 64. Available under license CC-BY-NC-4.0. Copyright 2022 Zheng, W. (b) Schematic of the experimental TERS setup and (c) TERS surface map, showing areas of higher and lower catalytic activity. Panels b and c are reproduced with permission from ref 62. Copyright © 2019 American Chemical Society. This publication is licensed under CC-BY-NC-ND. (d) TERS time evolution, showing chemical changes at one point on surface over time. After an initial hold at the onset potential of OER to observe a stable spectrum over time, the potential was switched to an OER potential. Panel d is reproduced with permission from ref 20. Copyright © 2019 American Chemical Society.

Tip-enhanced Raman spectroscopy (TERS) is a technique which allows Raman spectra of a surface to be acquired with nanometer resolution, by positioning the sample between a sharp probe and a Raman detector. Like SERS, it relies on the LSPR of nanoscale metal structures to generate an increased Raman signal but has the added benefit of providing morphological information as well. A sharp AFM-style tip with a rough silver coating locally enhances the Raman signal surrounding the tip, allowing lateral resolution of 1 nm or better to be obtained (Figure 5b). TERS can locally image the chemical and morphological structure of the surface, enhancing the signal by a factor of 10^6^ compared to Raman, and also gives a significant enhancement over SERS.^62,63^ One major downside to TERS besides the highly specialized equipment needed is the extensive setup required prior to measurement, and limited stability to the specially coated probe tip. Additionally, like other scanning probe methods, for acquiring an image of the surface the rate of scanning is limited by the hardware. To collect data more rapidly, catalysis at a selected point on the surface can be tracked, rather than scanning the entire surface. However, the limitations so far have prevented widespread adoption of TERS in catalysis. Kumar et al. demonstrated TERS using a zirconium oxide-coated silver scanning probe tip (532 nm) to acquire 500 nm x 500 nm 2D maps of catalytic activity (Figure 5c). The zirconium oxide coating enhanced tip stability, while the tip without the coating tends to have poor stability in aqueous conditions. For this example, the time to acquire the 2D map was dictated by the pixel size (10 nm) and the Raman signal integration time at each pixel (1s), which was around 40 min for one map.^62^ In addition to creating a 2D map, TERS can probe the evolution of a the Raman signal of a single point on the surface over time as demonstrated by Chen et al., and as discussed in EC-STM section (Figure 5d).^20^

Infrared (IR) spectroscopy complements Raman spectroscopy, because the selection rules for allowed IR transitions often differ from the ones for Raman transitions. By using these techniques together, a more complete picture of the material can be captured. Similar to Raman, IR is not surface specific, and additionally IR measurement at a liquid/solid interface is challenged by the fact that many electrolytes have a strong IR absorbance. Methods developed to address these issues include sum-frequency generation (SFG), Polarization Modulation IR Absorption Spectroscopy (PMIRS), Attenuated Total Reflection IR (ATR-IR), and Diffuse Reflectance Infrared Fourier Transform Spectroscopy (DRIFTS). For PEC and PC measurements, specialized cells can allow for illumination in addition to the probing IR beam (see the ATR-IR example below). Kemna et al. applied absorbance mode FTIR to a Pt CO_2_RR catalyst using an electrochemical cell with an IR-transparent window and an approximately 25 μm thick electrolyte layer, continuously measuring IR spectra with each spectrum averaged for 50 s. For measurements of the catalyst surface, to eliminate signals from the thin electrolyte layer, SFG was used. SFG selectively detects anisotropic transitions of surface components lacking inversion symmetry, enabling the identification of both a reaction intermediate in the solution phase (e.g., an ionic liquid component bonded to COOH) and adsorbates on the Pt surface. Since SFG revealed minimal CO adsorption on the Pt surface, it was proposed that a major CO_2_RR pathway progressed through the ionic liquid intermediate observed in the solution phase. These insights, unattainable with conventional IR alone, highlight SFG’s unique value in probing electrified interfaces with high chemical and temporal resolution (Figure 6a).^65^ In PMIRS, surface sensitivity is achieved by subtracting the signal component without a dipole perpendicular to the substrate plane, using alternating s- and p-polarized incident light. Recently PMIRS was used to measure the adsorption of CO on a Cu(100) CORR catalyst in an electrochemical cell. The emergence of an adsorbed CO stretching frequency at lower energy than free CO, along with its dependence on applied potential and electrolyte ions, provided insights into surface dynamics. The varying potential further isolated the surface signal, as it changed with potential, unlike signals from solution-phase species.^66^ Another IR method used to isolate surface signals in *operando* studies is ATR-IR, where the IR beam reflects multiple times through a crystal in contact with the sample, generating an evanescent wave that interacts with surface species. While the resulting signal is often weak, it can be enhanced using synchrotron sources or surface-enhanced IR absorption spectroscopy (SEIRAS) with rough metal substrates.^67^ For example, Zhang et al. used ATR-IR to probe O–O bond formation mechanisms on a hematite OER photoanode at different pH values. The authors modulated the potential applied to hematite in an electrochemical cell during illumination from the back through a transparent FTO substrate, while IR spectra were acquired from the front surface.^68^ Finally, DRIFTS complements these techniques by scattering the IR beam off the surface of a polycrystalline electrode. Lu et al. employed DRIFTS to measure proton adsorption on oxygen atoms in a TiO_2_ nanosheet catalyst substituted with atomically dispersed Pt, tracking the TiO-H signal’s magnitude as a function of applied potential. This provided direct observation of proton adsorption dynamics, further enriching the understanding of surface processes.^55^

**Figure 6:**
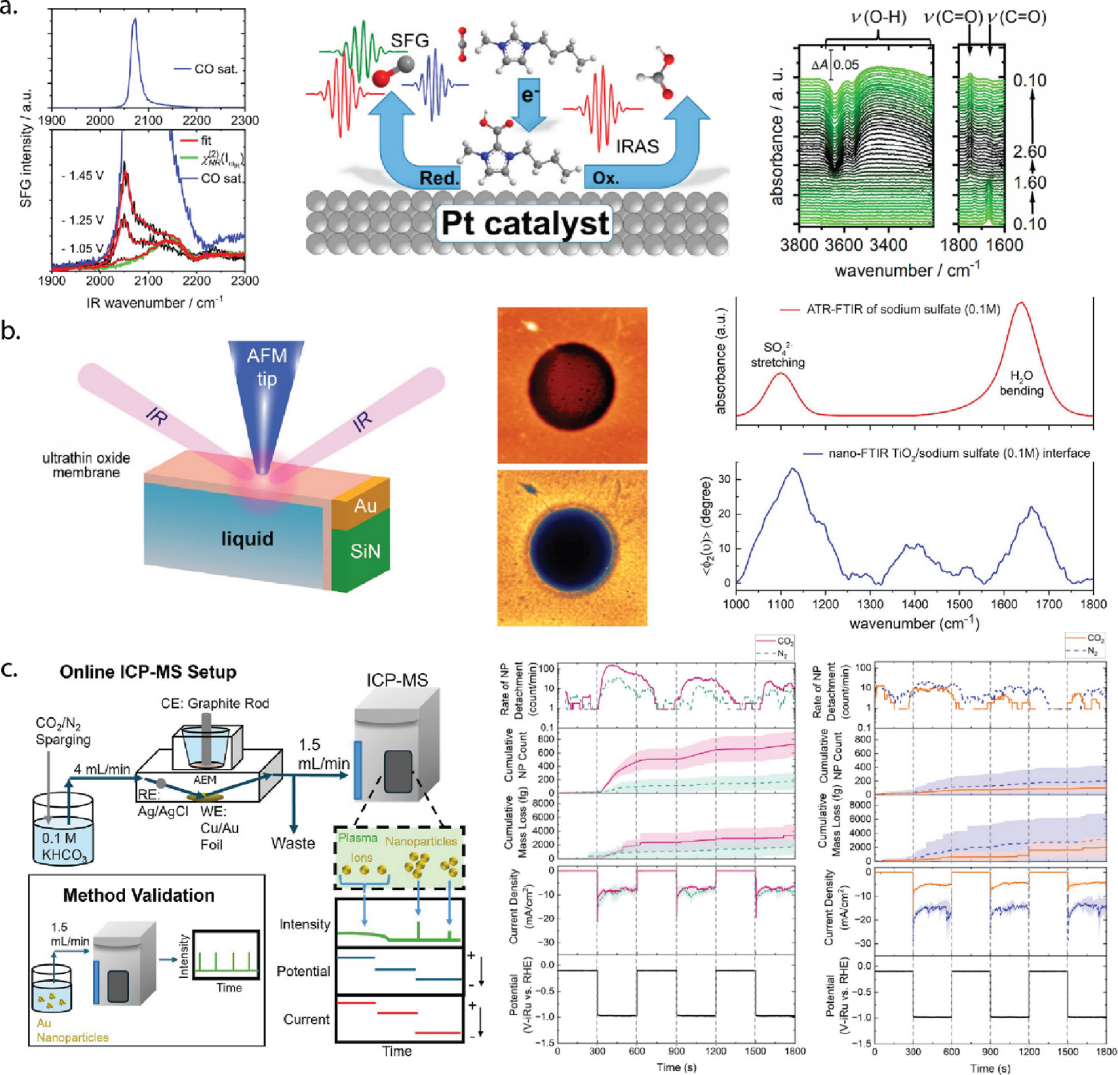
(a) Schematic and results of *operando* IR absorption, and SFG spectroscopy to address CO2RR at Pt electrode surfaces. Panel a is reproduced with permission from ref 65. Copyright © 2019 American Chemical Society. (b) Schematic of Plasmon-enhanced nano-FTIR setup with synchrotron IR. AFM topography and corresponding near-field optical (second-harmonic) map. Bulk ATR-FTIR spectrum (top) versus nano-FTIR spectrum (bottom) of the same solution. Panel b is reproduced with permission from ref 46. Copyright © 2020 American Chemical Society. (c) Schematic of online ICP-MS setup and method validation as well as the result for Au foil (red and green) and Cu foil (orange and purple). Nanoparticle detachment rate during 5 min steps at −0.1 V and −1.0 V vs RHE in CO_2_- and N_2_-saturated 0.1 M KHCO_3_, with corresponding cumulative mass loss, detached-particle count, current density, and applied potential were shown. Panel c is reproduced with permission from ref 70. Copyright © 2025 American Chemical Society.

While the previously mentioned *operando* IR methods lack morphology information, recently an approach to measuring localized IR signals using a scanning probe tip to plasmonically enhance the IR signal of nearby structures, thereby providing localized IR spectra in a proof-of-concept measurement. Using the nano-FTIR setup, the IR absorbance of a sodium sulfate solution in the region immediately adjacent to a TiO_2_ surface was measured through a thin TiO_2_/graphene membrane covered a liquid chamber containing the electrolyte. Additional peaks were seen in the plasmon-enhanced IR spectrum compared to an ATR-FTIR spectrum, resulting from modes only present in the electric double-layer adjacent to the surface because of the enhanced IR signal near the tip vs in the bulk. A scanning probe image of scattered IR light was also acquired, contrasting areas of greater or lesser absorption (Figure 6b).^46^ Methods such as this show promise for measuring chemical changes in solution in close proximity to a catalyst particle, for example, ordering of ions and molecules, similar to the observed double layer in this study, and for evolving concentrations of intermediates and products.

A final technique to quantify chemical changes during catalysis looks at the electrolyte. In-line Inductively coupled Plasma Mass Spectrometry (ICP-MS) during a chronoamperometry measurement was used to quantify Pt counter electrode dissolution over time in a model p-Si HER cell. *Ex-situ* studies had indicated that both Pt and Ag (from the reference electrode) deposited on the p-Si following operation, but it was unknown under which conditions Pt and Ag dissolved. Electrolyte concentrations of Pt were monitored by ICP-MS in-line with the HER flow cell. The beauty of this technique resides in monitoring the degree of catalyst dissolution as a function of changes in potential, while steady-state operation resulted in a much lower rate of dissolution. While there is a time delay caused by in-line measurement, this can be calibrated and accounted for in analysis.^69^ This powerful analytical approach has recently been extended to probe the complex degradation dynamics of catalysts in other critical energy reactions. By using online ICP-MS, Yan et al. revealed that catalytic material loss in Au and Cu catalysts occurs not just through ionic dissolution but also via the physical detachment of nanoparticles from the electrode surface (Figure 6c).^70^ In a complementary study, Pedersen et al. employed *operando* ICP-MS to uncover the crucial role of the local environment. They demonstrated that when Fe active sites dissolve, the subsequent consumption of protons during the reaction causes a sharp local pH increase, leading to the reprecipitation of the dissolved iron catalyst as oxide nanoparticles on the catalyst itself.^71^ Together, these works highlight a paradigm shift in using in-line ICP-MS, moving beyond merely quantifying material loss to elucidating the specific form of the lost material (ions vs nanoparticles) and its ultimate fate (escaping vs reprecipitating), thereby providing an unprecedented, mechanistic view of catalyst instability.

One final aspect of *operando* characterization relevant to solar-fuel generation is the direct measurement of the reaction kinetics and functional properties of the electrocatalyst while it is operating. One aspect of this is tracking bulk electrochemical performance, which is integral to most operando measurments. In the following, we present examples of methods that provide direct insight into catalyst function and kinetics, by measuring electronic or electrochemical changes in the surface during catalysis, often through a scanning probe format. One limitation to directly measuring functional properties in an *operando* electrochemical system with scanning probe techniques is interference of the electrolyte, which can either screen or conduct charges, but methods to insulate the bulk of the tip while maintaining a conductive channel have advanced greatly. Scanning electrochemical microscopy (SECM) measures redox currents close to the surface with an ultramicroelectrode, while surface-interrogation SECM (SI-SECM) is used to track reaction kinetics. Both methods bridge measurement of chemical species with functional electrochemical behavior and help elucidate complex catalytic mechanisms and processes. Electronic or electrochemical data itself can also be measured point-by-point under catalytic conditions using pc-AFM and PS-EC-AFM to provide maps of current or voltage superimposed on catalyst morphology. When illumination is required, SPM techniques employ a variety of methods, including fiber optic illumination from the side, illumination from the bottom utilizing a transparent electrode, or illuminating through the probe itself as in the next section to create photocatalytic conditions which are then probed.

SECM is a method based on SPM that measures redox currents generated at the sample surface by using a small (0.16–25 μm) ultramicroelectrode (UME) made of a thin wire encased in glass or another insulator. While there are many literature examples of in-house designed UMEs, commercial UMEs are also available. Many SECM setups are dedicated to that measurement, but since there can be challenges from topological signals convolving with the electrochemical signals, commercial and home-built combination AFM/SECM have also been created to simultaneously monitor morphology. In SECM, the UME is scanned or held close to the catalyst surface with constant or ramped potentials applied, with the UME as one working electrode, and the catalyst as the other. The current measured at the UME indicates the presence of the intermediate or product being studied as redox reactions occur near the UME. A redox couple is often used in the measurement. Additionally, it offers high sensitivity to electrochemical processes and can work in both aqueous and nonaqueous environments. However, disadvantages include the difficulty in separating electrochemical activity from topographic effects, which can complicate data interpretation. The technique also faces challenges in achieving atomic-level resolution due to the relatively large tip size and working distance. Furthermore, the presence of the tip can influence the local electrochemical environment, potentially altering reaction kinetics. Despite these limitations, SECM remains a valuable tool for understanding structure–activity relationships in electrocatalysis, especially when combined with other techniques like AFM or STM for enhanced functionality.

In one recent example with an interesting setup, a phosphorus-doped BiVO_4_ microcrystal was assessed for photocatalytic behavior using the UME as a light guide as well as a redox probe in a photo-SECM measurement. Because of the ability to spatially resolve chemical species data, the authors were able to directly observe OER and HER on different facets - (110) and (010), respectively - of the ∼3 μm microcrystal under illumination (Figure 7a,b). One key to this interesting result was the successful fabrication of 160 nm diameter Pt nanoelectrode, which was done using a laser puller and microforge. After the nanoelectrode was complete, it was polished and modified with ferrocene to facilitate detection of the products.^72^ A recent innovation that directly addresses the challenge of probing complex catalytic systems with multiple competing products is the development of sequential voltammetric SECM (SV-SECM) by Nam et al. This method was specifically designed to overcome a critical limitation where one reaction product, such as CO in the CO_2_ reduction reaction (CO_2_RR), poisons the UME probe, thereby preventing the simultaneous detection of other products like H_2_. The SV-SECM technique employs a rapid, three-stage voltammetric sequence at each measurement point: an initial anodic sweep to strip the poisoning adsorbate (CO) from the probe, followed by two distinct potential steps optimized for the selective oxidation of H_2_ and CO, respectively. This elegant approach enables the deconvolution of signals from competing reactions, allowing for the *operando* mapping of partial current densities for each product. By spatially correlating these selectivity maps with microstructural data, as demonstrated for CO_2_RR on polycrystalline gold, SV-SECM provides a powerful route to resolve facet-dependent structure-selectivity relationships that were previously obscured in complex reaction environments.^73^

**Figure 7:**
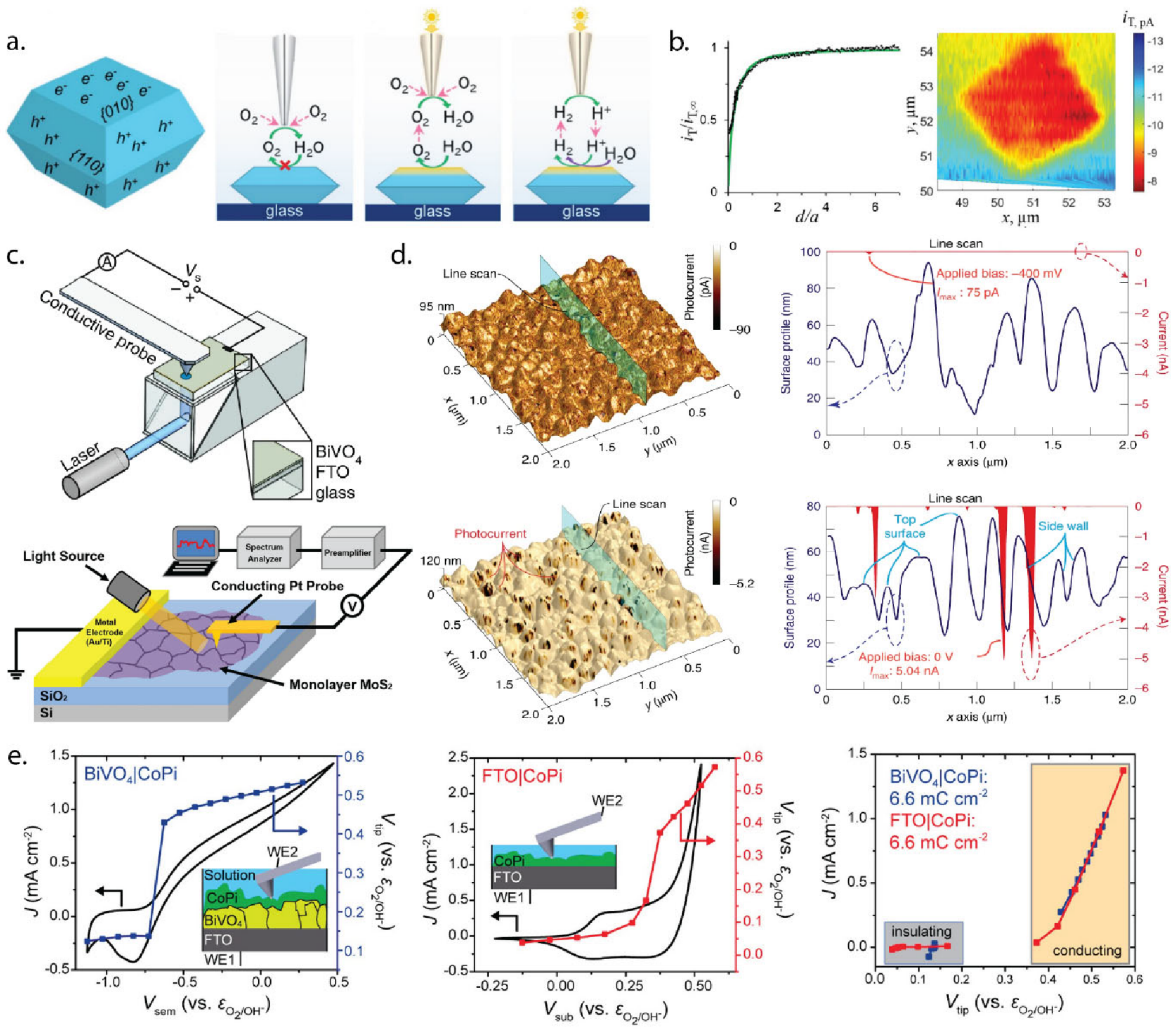
(a) Schematic representation of BiVO_4_ crystal and three types of SECM experiments. (b) ORR-based feedback-mode SECM experiments at a single P:BiVO_4_ microcrystal in the dark. Panels a and b are reproduced with permission from ref 72. Copyright © 2023 American Chemical Society. (c) Schematic illustration of the pc-AFM setup with different light source arrangements Panel c is reproduced with permission from refs 16, 87 . Available under license CC-BY-4.0. Copyright 2018 the authors. (d) PC-AFM characterization showing topography and corresponding surface profile and photocurrent extracted from the line scans for as-prepared (CA-0 h) and 10 h chronoamperometric test (CA-10 h) samples. Panel d is reproduced with permission from ref 6. Copyright © 2021, the authors, under exclusive license to Springer Nature Limited. (e) *Operando* PS-EC-AFM potential-stepping experiments: CV of CoPi-coated BiVO_4_ (black) with simultaneous tip potential Vtip (purple) under illumination, corresponding CV for CoPi-coated FTO (black) with Vtip (red) in the dark, and overlay of current density J and surface potential Vtip for BiVO_4_|CoPi (purple) under illumination and FTO|CoPi (red) in the dark. Panel e is reproduced with permission from ref 80. Copyright © 2018 American Chemical Society.

One often discussed use of SECM, particularly in combination with integrating with automation, is to assess an array of different materials sequentially, for example to find one with the lowest overpotential. This is particularly promising if the array consists of easily implemented progressions or variations on a theme, as shown by Mayer et al. for an array of three Sn/SnO_*x*_ catalysts for CO_2_RR to formate.^74^

A variation of the technique is Surface Interrogation SECM (SI-SECM), which is designed to quantify surface-adsorbed reaction intermediates and active sites. In this mode, a potential pulse is applied to the catalyst substrate to generate the species of interest. Subsequently, a titrant is electrochemically generated at the UME tip to react with and quantify these surface-bound species, providing direct insights into surface coverage and reaction kinetics. For example, a specialized configuration known as photoelectrochemical SI-SECM (PEC SI-SECM) was used by Kim et al. to probe the *operando* surface dynamics of water oxidation on a hematite (α-Fe_2_O_3_) photoanode. In this setup, a light pulse from the backside of the transparent catalyst generated photoexcited surface species, proposed to be Fe^4+^. After a controlled time delay, a titrant was generated at a gold UME tip positioned ∼4 μm away to quantitatively titrate these surface-bound intermediates, allowing for the direct quantification of the photoactive site density.^75^ More recently, Han et al. applied a potential pulse to a CuO substrate generating Cu­(III) active sites for OER, which were then titrated using a tip-generated redox mediator. By introducing controlled time delays before titration, the study also determined the kinetic rate constant of the reaction intermediates, showcasing the power of SI-SECM to deconstruct complex electrocatalytic mechanisms.^76^

*Photoconductive AFM (pc-AFM)* maps the current generated in a photoactive catalyst with high resolution by biasing a conductive AFM tip and measuring the current as the tip scans across the sample. Illumination can be from the backside of a transparent catalyst or from the side for others (Figure 7c), and the results show a correlation of photocurrent with defects, grain boundaries or specific crystal facets and other morphological structures. Because of the stray currents that would occur at the tip in an electrochemical cell, pc-AFM is typically performed in air or inert environments rather than in liquid electrolytes. However, pc-AFM is versatile, applicable to diverse photoactive materials such as semiconductors, perovskites, and thin films, and it provides quantitative insights by measuring absolute photocurrent values, enabling comparisons of charge-carrier generation and transport across regions or materials. On the other hand, pc-AFM also has limitations, such as its constraint to ambient conditions, which excludes direct study of electrochemical interfaces or hydration effects critical for catalysts in aqueous environments. Tip artifacts can also be an issue, as conductive coatings on tips may degrade, and tip–sample contact forces can perturb soft materials like organic photovoltaics. Furthermore, the throughput trade-off means high-resolution scans are time-consuming, limiting large-area or statistical analysis, and the simplified illumination may not replicate real-world solar spectra or device-level light absorption due to localized tip-based excitation. Despite these limitations, pc-AFM remains a powerful tool for resolving structure–activity relationships at the nanoscale, though complementary techniques such as *in situ* EC-AFM or SECM are often necessary for a more holistic understanding of catalyst behavior in realistic operating conditions.

In one of our studies, we used pc-AFM to measure the effect of metal oxides on the stability of BiVO_4_ OER photoanodes. Metal oxides or other catalysts were proposed to improve stability by transporting charges away from the surface. It was seen that by depositing a charge transporting metal oxide (Co_0.4_Fe_0.1_Ce_0.5_Ox) on BiVO_4_ surface, followed by a catalytic metal oxide (Ni_0.8_Fe_0.2_Ox), charges were transported through the material and to the surface effectively, thus improving stability.^77^ In a subsequent study, we showed that pc-AFM can resolve the morphologic features leading to increased photocurrent in a ‘self-improving’ GaN HER catalyst. We illuminated the GaN samples from the side and measured current from the surface before and after 10 h of catalysis. Significant increases in the photocurrent generated at the GaN side walls, compared to top facets, were seen following the self-improvement (Figure 7d). Further work with *ex-situ* STEM-EELS revealed through elemental analysis of the samples that self-improvement generated gallium oxynitride on the side walls over time during operation, suggesting the mechanism for the protective nature being the formation of gallium oxynitride.^6^ More recently, by using pc-AFM, we correlated the Zn-rich regions in ZnTe photocathodes with localized photocurrent enhancement, revealing the critical role of surface composition in optimizing photoelectrochemical CO_2_RR performance.^78^

*Potential-sensing electrochemical AFM (PS-EC-AFM)* is a SPM technique that has been developed in the past few years to measure the (photo)­voltage of a working electrode in an active electrochemical cell. A commercial insulated AFM tip is used, which has a conductive 25 nm radius apex and insulating side walls to prevent extraneous currents. Morphology of the catalyst is first assessed, followed by moving the tip to and capturing surface (photo)­voltage of particular areas under electrochemical conditions. The surface potential or photovoltage of the catalyst is determined from the applied potential and the measured potential at the tip. The PS-EC-AFM technique was first demonstrated to study the role of cobalt (oxy)­hydroxide phosphate (CoPi) as an OER catalyst on planar and mesostructured hematite (α-Fe_2_O_3_) photoanodes. A custom electrochemical AFM cell combined bottom illumination of the photoanode and precise potential sensing via the AFM tip. This new technique uniquely enabled the direct, nanoscale measurement of the surface potentials during operation, revealing local variations across heterogeneous or mesoporous surfaces that are inaccessible to conventional methods. It identified the conductivity switch in CoPi, showing that it transitions to a conductive state at specific substrate potentials and acts as both a hole collector and an oxygen evolution catalyst. Additionally, it quantified potential drops within catalyst films and correlated the catalyst surface potential with photovoltage, providing detailed insights into the mechanisms driving water oxidation in photoelectrochemical systems (Figure 7e).^79,80^

More recently, PS-EC-AFM was used to measure charge transfer at the interface between Ni OER catalyst nanoparticles and a n-Si photoanode substrate to determine the effects of particle size on photovoltage and selectivity of charge transfer. A greater photovoltage as well as the selective transfer of holes into the electrolyte from the smaller catalyst particles was seen but absent in *ex-situ* conditions.^81^ While PS-EC-AFM provides unique information about the catalyst, it has challenges including those known for AFM (e.g., uniformity of imaging in fluid, slow scan speeds, and small imaging areas as well as relative intolerance for large step heights).

Increasing complexity of the solar fuel electrocatalytic surfaces for improved efficiency and selectivity demand enhanced *operando* analytical techniques, to explore morphology, chemical species, and functional properties of the catalyst. Dual site and dual function catalysts have arisen as holding particular promise because they can enhance reaction efficiency and selectivity while decreasing cost,^55,82,83–85^ and incorporation of a charge transfer catalyst has been seen to speed the reaction by improving kinetics.^86^ Catalysts that decreased cost by dispersing elements such as Pt over a larger area has been shown to have better catalytic activity than commercial Pt/C catalysts, suggesting that while attempting to address cost (or efficiency) by generating creative new catalytic designs, the other concern could be solved as well as better “atomic efficiency” is obtained.^55^ Design Of Experiments, Machine Learning/AI and robotics have been shown to provide a framework for speeding material selection, synthesis, and analysis, promising large libraries of proposed materials to analyze in the future yet needing systematic *operando* methods to evaluate their promise for real world application. To assess these and other next-generation solar fuel catalysts, simultaneous collection and correlation of functional, structural, and/or chemical data with electrochemical performance is needed to fully understand the multiple complex interactions at play. More widespread application of data analysis and artificial intelligence/machine learning (AI/ML) methods are needed to fully exploit the large amounts of data from *operando* imaging to guide experiments and extract new meanings from the results.

Looking forward, several critical considerations must be addressed to ensure the reliability and relevance of *operando* characterization techniques. The presence of probing elements - whether electromagnetic radiation, electron beams, or physical tips - can potentially perturb the electrochemical interface, creating conditions that deviate from real operating environments. This challenge becomes particularly complex in photoactive systems, where both sample illumination and measurement probes must be carefully managed. For instance, in electron microscopy studies of photocatalysis, distinguishing between photon-induced and electron beam-induced effects requires careful experimental design, as high-energy electrons can trigger processes that would not occur under photon illumination alone.^17^ These considerations necessitate thoughtful optimization of multiple parameters: beam intensity, scanning speed, tip–sample interactions, optical paths for homogeneous illumination, and cell designs that minimize dead volume while enabling effective sample analysis. Furthermore, the challenge of reducing interference from measurement conditions (such as solvent effects in FTIR or beam perturbation in EM) while maintaining measurement sensitivity has driven continuous innovation in cell design and measurement protocols.^87^ To address these challenges, researchers increasingly employ cross-validation through multimodal characterization approaches and comparison with *ex-situ* studies, which becomes essential to verify the accuracy of *operando* observations and distinguish genuine electrochemical processes from measurement artifacts. Such comprehensive validation strategies, combined with thoughtfully designed experimental setups, will be crucial for advancing our understanding of dynamic interfacial processes in electrochemical systems.

AI and machine learning (ML) have recently been used to develop high-throughput *operando* imaging platforms,^88,89^ to optimize,^90^ and to extract new insights from data.^91,92^ This is only a highlight of the field, and extensive discussions of AI/ML integration into *in-situ*/*operando* experiments may be found in recent reviews, including Chen et al.^93^ and Li et al.^94^

Advances in robotic and automated laboratories have led to streamlined experimentation^90,95^ and through correlating automated data generation with real world solar fuel device performance of the catalysts, closed-loop laboratories can deliver real results. Robotics can also greatly improve the reproducibility of electrochemical measurements, enhancing collaborative efforts.^96^ However, aligning to the concerns in previous discussion, the full potential of these technologies can only be realized through standardized and collaborative data-sharing practices. Efforts are now underway to establish standardized protocols and formats for sharing complex electrochemical and spectroscopic measurements, including polarization curves under specific conditions (e.g., substrates and pH). This standardization is critical for ensuring reproducibility, facilitating cross-institutional validation, and accelerating the development of innovative catalytic materials and processes. A key aspect of this initiative involves the standardization of cell design and experimental setups, which are often variable across laboratories. Uniform geometries, electrode configurations, and electrolyte compositions reduce variability, while detailed metadata sharing ensures accurate replication. This transparency enhances data reliability and fosters collaborative research by establishing a common framework. Collaborative platforms could also incorporate AI-driven tools to analyze and cross-reference data sets, identifying trends and generating new hypotheses. By prioritizing data sharing and standardization, the field of catalysis can move toward a more open and interconnected research ecosystem, ultimately driving innovation and advancing our understanding of complex catalytic interfaces.

Computational methods have become vital to process and parse data coming from *operando* imaging of increasingly complex catalysts, and methods such as DFT and molecular dynamics will become even more important in modeling them. Many *operando* experiments discussed here used simulations or advanced data analysis techniques to interpret results. Experimental results combined with theoretical calculations can be used to create a ‘digital twin’ that will allow full exploitation of the predictive power of the collected simulation and correlative data. DFT calculations suggested that increasing the coverage of adsorbed CO would result in better ethanol selectivity by Cu nanoparticles, which led Li et al. to add FeTPP­[Cl], a known CO_2_ to CO catalyst, as a cocatalyst to Cu nanoparticles. The cocatalyst addition substantially enhanced ethanol production, demonstrating the importance of incorporating computation with experiment.^56^

Current *operando* analysis faces significant obstacles when studying inaccessible interfaces, such as bubble interference, buried heterojunctions, solid-electrolyte interphases, or integrated microelectronic systems. These challenges stem from the difficulty in achieving high spatial and temporal resolution simultaneously, especially under dynamic operational conditions (e.g., high temperatures, voltages, or chemical reactivity). Additionally, the presence of complex, multilayered structures often obscures signals, making it hard to isolate and interpret interfacial phenomena. To overcome these limitations, future advancements could focus on sample modification and developing hybrid techniques that combine complementary methods to enhance resolution and sensitivity. Furthermore, leveraging machine learning for real-time data analysis and modeling could help deconvolute complex signals, while standardized protocols and open-access databases would facilitate cross-validation and reproducibility. By addressing these challenges, *operando* analysis can unlock deeper insights into hidden interfaces, driving innovation in energy, electronics, and beyond.

There are limitations to *operando* imaging, which require resolution through technological advances. For example, *operando* Kelvin Probe Force Microscopy (KPFM) has not been attainable in a liquid phase because the KPFM signal depends on the interaction of electric fields at the surface affecting the motion of the scanning probe tip. By covering the surface with electrolyte, the charges on the surface are screened, damping the signal.^97^ On the other hand, *operando* surface voltage has recently been obtained by advances in PS-EC-AFM, a method which shows promise for future application to catalytic development. Vibrational spectroscopy methods can generate heat around the sample location, shifting the catalytic surface out of *operando* conditions. To assess these risks for any experiment, simulations can be used to determine the order of magnitude of effect this will have on the spectrum. Computational methods can also assist with overcoming fundamental limitations by some of those methods. For example, SECM is limited in resolution for imaging a surface by the slow diffusion of surface species to the probe. However, through use of new image processing algorithms, the measured signal is deconvoluted into the true point signal and the point spread function, permitting significant advancements in resolution for a given SECM probe.^98^

Scanning ion conductive microscopy (SICM) measures morphology, surface charge, and ion flux, and could be applied to spatially map the strength of electrolyte ion interactions with, and interfacial capacitance of, a catalyst. It was recently applied *ex-situ* to determine the interfacial capacitance on a layered graphene oxide (GO) surface under electrolyte, as well as the anisotropic permeability of GO to the different ionic species.^99^

SPM techniques can be developed to facilitate application to more *operando* situations. For example, while there are no fundamental limitations to most SPM techniques under a layer of electrolyte, there are technical difficulties including visible contaminants in the electrolyte, evolution of bubbles, and rapid evaporation of electrolyte droplets in some atmospheres over long measurements. Recently, a long-duration SPM electrochemical corrosion study used a 2 μm micropipette to form a map of lower vs higher stability regions of an aluminum surface. To prevent evaporation of the electrolyte layer, the surface was covered by a layer of oil, with small electrolyte droplets on the surface under the oil in a matrix. Open circuit potential (OCP) at each location was measured for 100 s. Oil immersion experiments like this could allow the use of arbitrary ambient humidity levels and electrolyte. This method could also allow a smaller electrolyte droplet and higher resolution, because the contact angle of electrolyte under oil is higher than in air.^100^

In OER, HER, or other gas evolving catalysts, bubble formation can affect the SPM measurement, simply due to the small sizes of the probe and the area being examined. A common way to address this is to hold the potential of the catalyst near the reaction onset potential, rather than in the current saturation regime, which prevents generation of large bubbles while still observing the system under operating conditions. This may result in less representative *operando* conditions, however. Recently it was found that, at least in some cases, clear images may be obtained at potentials close to the operating potential rather than close to the onset potential. The images were obtained despite the increased gas evolution, indicating that there are a variety of parameters to adjust in these situations to get good quality images.^23^ Finally, recent improvements to technology facilitate probe tuning and the stable measurement of liquid SPM.^23^ Future work is sure to build on these developments.
